# The partitioning of TCR repertoires by thymic selection

**DOI:** 10.1084/jem.20230897

**Published:** 2024-08-21

**Authors:** Wan-Lin Lo, Eric S. Huseby

**Affiliations:** 1 Division of Microbiology and Immunology, Department of Pathology, University of Utah School of Medicine, Salt Lake City, UT, USA; 2 Department of Pathology, University of Massachusetts Chan Medical School, Worcester, MA, USA

## Abstract

αβ T cells are critical components of the adaptive immune system; they maintain tissue and immune homeostasis during health, provide sterilizing immunity after pathogen infection, and are capable of eliminating transformed tumor cells. Fundamental to these distinct functions is the ligand specificity of the unique antigen receptor expressed on each mature T cell (TCR), which endows lymphocytes with the ability to behave in a cell-autonomous, disease context–specific manner. Clone-specific behavioral properties are initially established during T cell development when thymocytes use TCR recognition of major histocompatibility complex (MHC) and MHC-like ligands to instruct survival versus death and to differentiate into a plethora of inflammatory and regulatory T cell lineages. Here, we review the ligand specificity of the preselection thymocyte repertoire and argue that developmental stage–specific alterations in TCR signaling control cross-reactivity and foreign versus self-specificity of T cell sublineages.

## Teaser

TCR rearrangement endows the preselection thymocyte repertoire with wide-ranging ligand specificity. Here, we discuss how thymic selection matures or eliminates clonotypes with particular specificities and develops T cell sublineages and microrepertoires according to their antigen-recognition capabilities.

## Introduction

**Figure 1.**
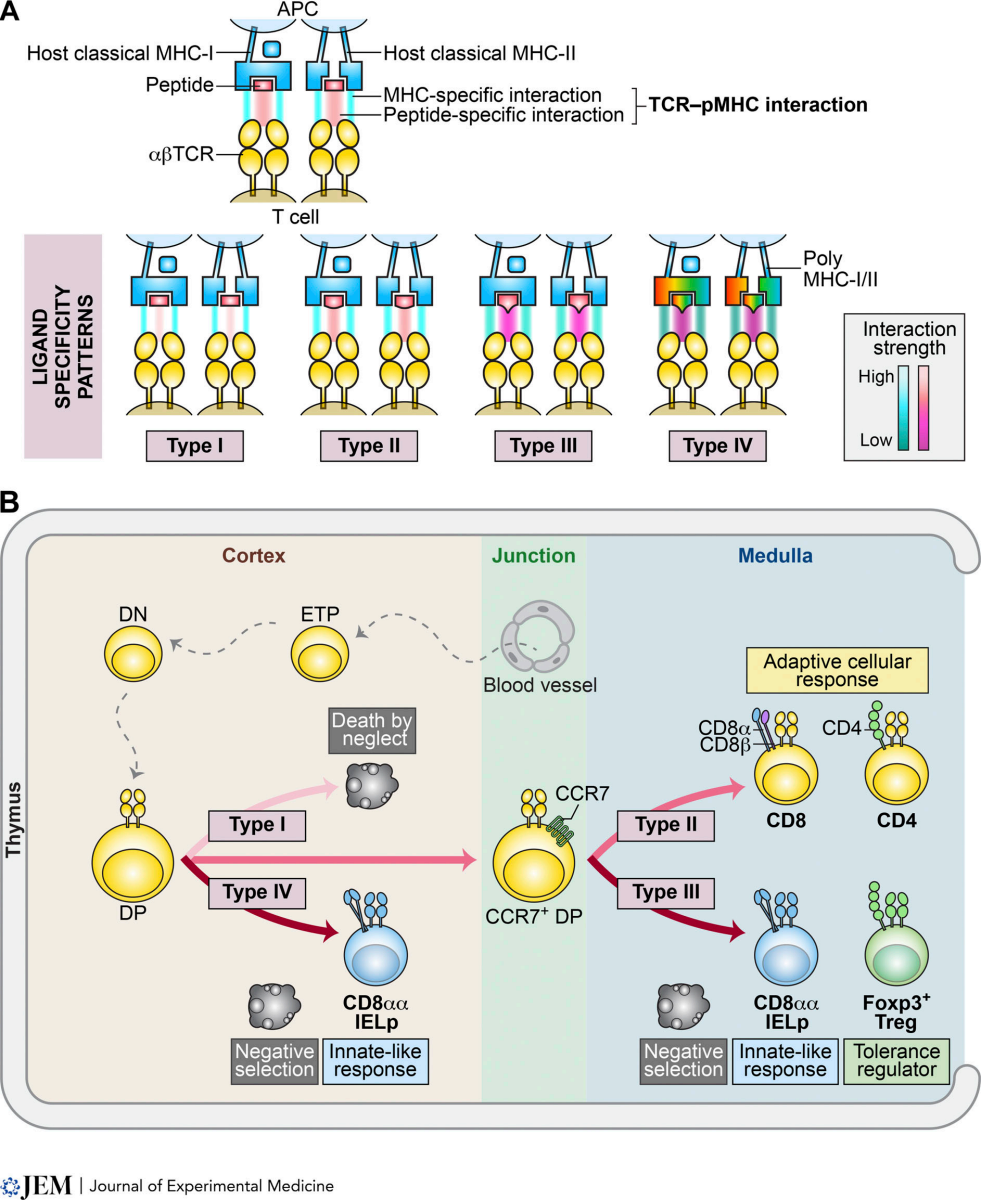
**Post-selection T cell subsets and their TCR ligand specificity patterns. (A and B)** An overview of the four distinct patterns of TCR–pMHC ligand interactions (A) and the categorization of post-selection αβ T cells into three groups (B). **(A)** The ligand interactions are classified as non-reactive (Type I), weakly self-reactive (Type II), and strongly self-reactive (Type III and IV). The illustration highlights the interactions between MHC and TCR molecules (top panels), where blue lines indicate the common amino acid–mediated interactions (specific to MHC), and red lines represent the peptide-mediated interaction (peptide specific). These interactions together determine the overall TCR–pMHC interaction strength. Importantly, Type IV interactions are often characterized by their potential to crossreact with multiple MHC alleles (poly MHC-I/II). **(B)** The three subsets of αβ T cells comprise: (1) naive CD4 or CD8 T cells (Type II ligand specificity pattern), each distinguished by their unique functional roles; (2) Treg cells (Type III pattern), which play a crucial role in initiating and sustaining immune tolerance; and (3) CD8αα IEL precursors (Type III or IV patterns; IELp), characterized by their ability to respond rapidly without the need for TCR activation. It is important to also note that CD8αα IELp typically lack CD8α expression initially but will re-express the CD8αα co-receptor in the peripheral tissues. ETP: early thymic precursor cells.

T cells circulate throughout the body, alert for signs of pathogen infection or tumorigenesis. The ability of T cells to distinguish healthy and diseased cells arises from their expression of an antigen receptor (TCR) that recognizes specific ligands presented by classical major histocompatibility complex (MHC) molecules, non-classical MHC molecules, and MHC-like proteins. For αβ T cells, these MHC-bound ligands typically consist of peptides ranging from 8 to 15 amino acids in length (Fig. 1 A). Additionally, certain specialized subsets of T cells can interact with presented lipids and small metabolites. Given that classical MHC molecules are the most polymorphic human genes (Trowsdale and Knight, 2013), individual T cell repertoires must undergo a “learning” process during T cell development to discern self from non-self, involving the recognition of both MHC molecules and their bound peptides.

Reflecting their functional diversity, αβ T cells departing the thymus can be loosely categorized into three groups (Fig. 1 B): naive αβ T cells, which exhibit effector functions and tissue-specific localization upon encountering their cognate antigens; regulatory T (Treg) cells, including Foxp3^+^ Treg cells, that play roles in anti-inflammatory processes and the maintenance of homeostasis; and “innate-like” T cells, such as CD8αα intraepithelial lymphocytes (IELs), which are attuned to barrier stress and dysbiosis. Within these subsets, naive αβ conventional T cells (Tconv) typically derive homeostatic signals from weak affinity interactions with antigen-presenting cells (APCs) that display self-peptides on MHC molecules (self-pMHC). In contrast, regulatory and innate-like clones exhibit higher levels of self-reactivity. Here, we discuss the ligand specificity of the preselection thymocyte repertoire and how subsequent thymic selection processes sculpt TCR features in T cell sublineages and microrepertoires according to their antigen recognition capabilities.

## T cell tolerance: Historical perspective

Historical studies have revealed that immune tolerance within the T cell compartment, which is essential to limit autoimmune disease, operates through host MHC restriction and TCR clonotype diversity and specificity (Burnet, 1976; Lederberg, 1959; Sell and Gell, 1965; Talmage, 1957; Zinkernagel and Doherty, 1974). The diversity of MHC alleles within a population serves as a barrier to pathogen evasion of immune responses (Kosmrlj et al., 2010; Messaoudi et al., 2002). In addition, individual T cell repertoires are trained to differentiate self from non-self during the process of thymic selection (Berg et al., 1989; Bevan, 1977; Kappler et al., 1987). Successful selection renders mice capable of receiving tissue grafts from syngeneic donors while rejecting those from donors with significantly different major or minor alloantigens. Major alloantigens are attributable to variations in MHC alleles, and minor alloantigens arise from differences in sex-specific proteins and non-MHC protein sequence polymorphisms (Billingham et al., 1953; Cudkowicz and Rossi, 1972; Owen, 1945).

In a theoretical model, Niels Jerne rationalized this phenomenon by proposing that T cells (or TCRs) are genetically predisposed to recognize MHC molecules (Huseby et al., 2004; Jerne, 1971). Drawing parallels with bacterial genetics, he proposed that the error-prone nature of DNA replication during vigorous cell proliferation in the thymus might introduce mutations in the TCR, potentially curtailing undue reactivity to host MHC molecules. While not all of Jerne’s mechanisms have been directly proven, the idea that TCRs are innately biased to interact with MHC ligands persists (Marrack et al., 2008). His theory accounts for why 10–20% of preselection thymocytes respond to host MHC ligands, with about half of these clones integrating into the mature T cell repertoire and the rest being eliminated by negative selection in the thymus (Huseby and Teixeiro, 2022). Our current knowledge of T cell development and how it confers the ability to distinguish self from non-self was greatly molded by this early perspective.

The evolution of the T cell repertoire, driven by the random generation of TCR sequences and filtered by selection rather than deliberate design, prompts a hypothetical question: Does incomplete negative selection offer an immunological benefit? This question dovetails with the motivation behind numerous studies that have shown negative selection to be imperfect and not wholly exhaustive. The formation of the TCR repertoire, which utilizes the randomness of somatic TCR gene segment recombination, must prepare the immune system for a broad spectrum of potential pathogen encounters over an individual’s lifetime. This approach evolved to build a large anticipatory T cell repertoire while minimizing the risk of autoimmune diseases. Therefore, each individual must develop and maintain a T cell repertoire, which balances “tolerance to self” and “effective discrimination of self from non-self.” Indeed, early research utilizing superantigen models and TCR transgenic mice yielded significant findings: the former studies showcased efficient deletion of thymocytes expressing specific Vβ chains in mouse strains harboring corresponding endogenous superantigens (Blackman et al., 1990; Kappler et al., 1987), and the latter illustrated antigen-specific clonal deletion occurring during the double-positive (DP) stage of thymocyte development (Kisielow et al., 1988; Sha et al., 1988). As a result, the concept of tolerance to self has long been intertwined with the “near perfect” effectiveness of thymic negative selection (Burnet, 1961, 1991; Hogquist et al., 1994). However, eliminating all self-reactive TCR clones could inadvertently produce gaps in foreign antigen specificity and preclude the creation of immunomodulatory regulatory and innate-like T cells, underscoring the complex task of immune system optimization (Davis, 2015; Vrisekoop et al., 2014).

## All TCRs are not created equal

Initially, stem cells populate the thymus and start differentiating into T cell lineage precursors, a process initiated by Notch signaling and characterized by the restructuring of transcriptional gene networks (Hosokawa and Rothenberg, 2020; Shin et al., 2024). This differentiation includes the induced expression and usage of key signaling enzymes and scaffolds, as well as RAG-mediated TCRβ recombination and β-selection events. These steps, followed by TCRα rearrangement, lead to the generation of preselection thymocytes, which depend on TCR signals for their survival and further differentiation (Davis and Bjorkman, 1988; Oettinger et al., 1990; Schatz et al., 1989; von Boehmer and Fehling, 1997). Each thymocyte expresses thousands of TCRs carrying an identical sequence on its surface, contributing to a preselection thymocyte repertoire that includes several million unique clonotypes (Arstila et al., 1999; Bradley and Thomas, 2019; Robins et al., 2010). Despite the complexities of early T cell development, αβ TCR^+^ preselection CD4^+^CD8^+^ DP thymocytes show uniform transcriptional and epigenetic characteristics (Chopp et al., 2020). This stage-specific consistency allows the specificity of the randomly generated TCR to dictate whether individual clones advance through the processes of positive selection and co-receptor choice or are eliminated (Itano and Robey, 2000).

T cell development encounters multiple conundrums while generating host MHC-restricted mature T cell repertoires. Rearrangement must yield a diverse set of TCRs capable of recognizing ligands presented by the myriad of MHC class and allele combinations in a species. While certain TCR variable (V) genes are predominantly expressed in either CD4 or CD8 T cells (Sim et al., 1996), no V gene segment is entirely excluded from forming receptors that can engage with structurally diverse MHC class I (MHC-I) or II (MHC-II) molecules (Garman et al., 1986). Consequently, individual TCRα or TCRβ chains can form parts of receptors that interact with a broad spectrum of polymorphic MHC ligands. However, αβ TCR pairs found on post-selection T cells are typically restricted to specific MHC alleles. Moreover, despite the millions of TCR sequences present in mature T cell repertoires, this clonal complexity cannot ensure comprehensive pathogen recognition if each T cell is confined to recognizing just one pMHC combination.

In other terms, there exist ∼5 × 10^11^ potential combinations for a nine-amino-acid peptide, alongside thousands of MHC-I and MHC-II alleles. Thus, TCR rearrangement and subsequent thymic selection of Tconv cells need to equip thymocytes with TCRs that can recognize various unique, foreign-derived peptides presented by host MHC molecules, thereby eliminating voids in the repertoire (Morris and Allen, 2012; Vrisekoop et al., 2014). To achieve “universal recognition” of peptides and MHC alleles, preselection thymocytes generate TCRs with diverse specificities for MHC and peptide residues through somatic recombination of V(D)J gene segments, nucleotide additions and deletions, and the pairing of randomly generated TCRα and TCRβ chains (Davis and Bjorkman, 1988; Lefranc, 2011). This process introduces randomness into the TCR antigen-binding site: a mosaic of V(D)J junctional amino acids (CDR3 segment) at its core, encircled by germline-encoded (CDR1 and CDR2) residues. This construct, together with the semiconserved diagonal TCR–pMHC binding orientation, favors engagement of CDR1 and CDR2 residues with MHC molecules and interaction of CDR3 residues with peptide residues (Garcia et al., 1996; Rossjohn et al., 2015). The incorporation of specific amino acids in the CDR3 and the effect of TCRα/TCRβ pairings enrich TCRs with a vast range of binding proficiencies to diverse foreign and self-pMHC ligands, often with relatively strong affinity (Chakraborty and Weiss, 2014; Huseby and Teixeiro, 2022).

At one end of the cross-reactivity spectrum, a TCR may recognize a wide array of distinct peptides presented by multiple MHC-I and MHC-II molecules. This broad recognition can occur when TCR residues are particularly responsive to the invariant features of MHC ligands, including the peptide backbone and conserved MHC residues, and show minimal interaction with the side chains of the amino acids of the bound peptide (Dai et al., 2008; Huseby et al., 2005; Stadinski et al., 2011). At the other extreme, there are TCRs generated that bind very few, if any, pMHC molecules, a likelihood that increases when the central CDR3 residues include specific amino acids like lysine and glutamic acid. In a more moderate scenario, TCRs can form high-affinity bonds with a select group of foreign or self-pMHC molecules, a capacity that correlates with the hydrophobic nature of the central CDR3 residues (Stadinski et al., 2016). For simplicity, TCRs can be grouped into four ligand specificity patterns (Fig. 1 A): (1) type I: non-pMHC reactive, (2) type II: weakly self-reactive with significant affinity for certain foreign pMHC ligands, (3) type III: strongly self-reactive, recognizing particular self-peptides presented by host MHC molecules, and (4) type IV: strongly self-reactive, capable of recognizing multiple self-pMHC ligands. Ultimately, thymic selection processes are essential to eliminate non-reactive and overly self-reactive TCRs, while effectively organizing those from categories 2, 3, and 4 into specialized microrepertoires, each tailored with distinct functionality.

## Thymocyte auditioning for selection

### Requirement for productive TCR signaling

Positive selection and T cell homeostasis together address the challenge of building the T cell repertoire. Beginning with preselection thymocytes in the thymic cortex, clones lacking appreciable reactivity to self-pMHC molecules fail to elicit substantial TCR signals and consequently undergo apoptosis due to neglect (Berg et al., 1989). This self-referential requirement persists throughout subsequent phases of thymic development and extends to peripheral T cells (Liu et al., 2003; Saini et al., 2010; Surh and Sprent, 2008). Theoretically, if a TCR can sufficiently recognize self-pMHC molecules above a specific affinity threshold, it implies a potential to also recognize a fraction of the vast array of possible foreign peptides—approximately 5 × 10^11^—bound to self-MHC molecules. Nevertheless, to mitigate the risk of autoimmunity, mature thymocytes and peripheral T cells implement strategies to fine-tune TCR signaling. Such regulation allows DP thymocytes to leverage weak self-pMHC interactions to induce their differentiation, while mature Tconv cells depend on these same interactions for survival signals (Hogquist and Jameson, 2014; Huseby and Teixeiro, 2022; Surh and Sprent, 2008; Yagi and Janeway, 1990).

**Figure 2.**
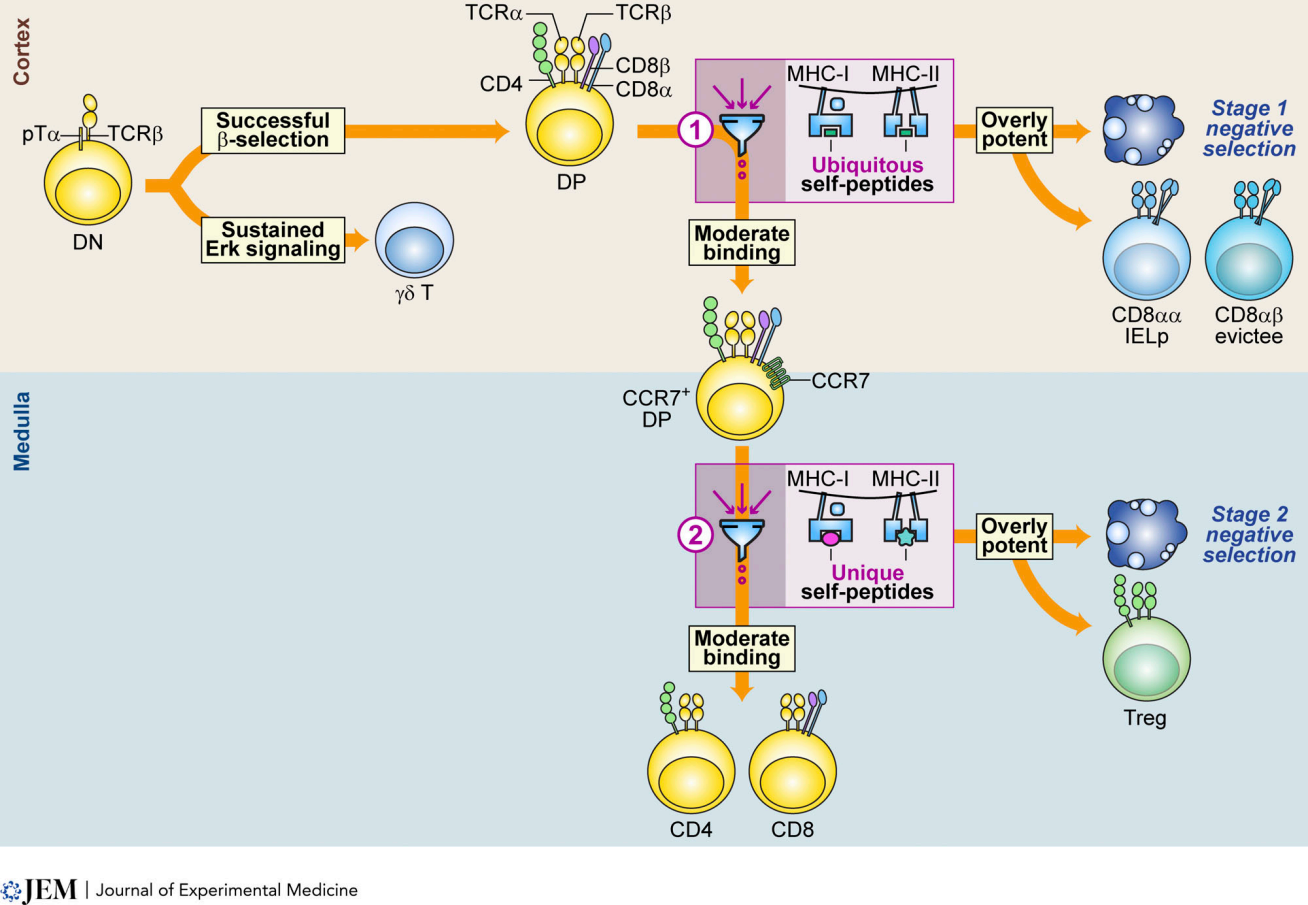
**Two-stage negative selection process and diverse outcomes.** The illustration outlines the two-stage negative selection process during thymic development. Initially, CD4^−^CD8^−^ DN thymocytes expressing pre-TCRα (pTα) and TCRβ undergo β-selection; successful thymocytes then progress to expressing the co-receptors CD4 and CD8, becoming DP thymocytes, and initiate TCRα rearrangement. The rearranged TCRα and TCRβ are subjected to thymic selection in the cortex and medulla, each stage serving a distinct purpose to filter and permit only appropriate TCRs to pass the selection checkpoints, depicted as two filter symbols (1 and 2). The initial screening in the cortex allows preselection TCRs to interact with ubiquitous self-peptides presented by cortical thymic epithelial cells. Only those TCRs demonstrating moderate affinity are allowed to pass the selection checkpoint, subsequently upregulating the chemokine receptor CCR7, and migrating to the medulla for the subsequent stage of thymic selection. Thymocytes with insufficient binding undergo death by neglect, whereas those with excessively strong binding are eliminated through negative selection (stage 1) or are redirected to become CD8αα IEL precursors (IELp). Cells signaling with excessive potency may also be expelled from the thymus and become evictees. Overall, the objective of stage 1 negative selection is to eliminate TCRs that exhibit broad cross-reactivity with multiple MHC molecules. Similarly, CD8αα IEL precursors, which are salvaged from this phase of negative selection, frequently demonstrate cross-reactivity toward various MHC molecules. Next, in the medulla, the ongoing selection process screens TCRs against tissue-specific antigens. Only those with moderate affinity are allowed to mature into CD4 or CD8 cells and egress to the periphery. On the other hand, TCRs with excessively strong binding undergo negative selection (stage 2) or are alternatively selected to become Treg cells. The aim of stage 2 negative selection is to eliminate TCRs that demonstrate overly strong reactivity toward tissue-specific self-peptides. Correspondingly, Treg cells, spared from this stage of negative selection, exhibit a higher degree of self-reactivity compared with conventional CD4 and CD8 T cells, often characterized by elevated expression levels of CD5 and Nur77.

The ability of thymocytes to navigate positive and negative selection processes based on the strength of TCR signaling against self-pMHC ligands is a hallmark of TCR signaling scalability. Thymocytes can be positively selected through weak affinity interactions with self-pMHC ligands, whereas more potent ligands trigger negative selection. This process is nuanced and includes developmental stage-specific responses: at the immature DP stage, cells that recognize self-antigens can undergo apoptosis (stage 1 negative selection) or develop into innate-like T cells (e.g., CD8αα IELs). In contrast, recognition at later developmental stages can result in apoptosis (stage 2 negative selection) or the emergence of Treg cell populations (e.g., Foxp3^+^ Treg cells), illustrating the dynamic nature of T cell development and the critical role of TCR signaling intensity and timing in determining cell fate (Cheroutre et al., 2011; Daley et al., 2017; Hogquist and Jameson, 2014; Huseby and Teixeiro, 2022; Josefowicz et al., 2012; Sprent and Kishimoto, 2002; von Boehmer and Kisielow, 2006) (Fig. 2).

**Figure 3.**
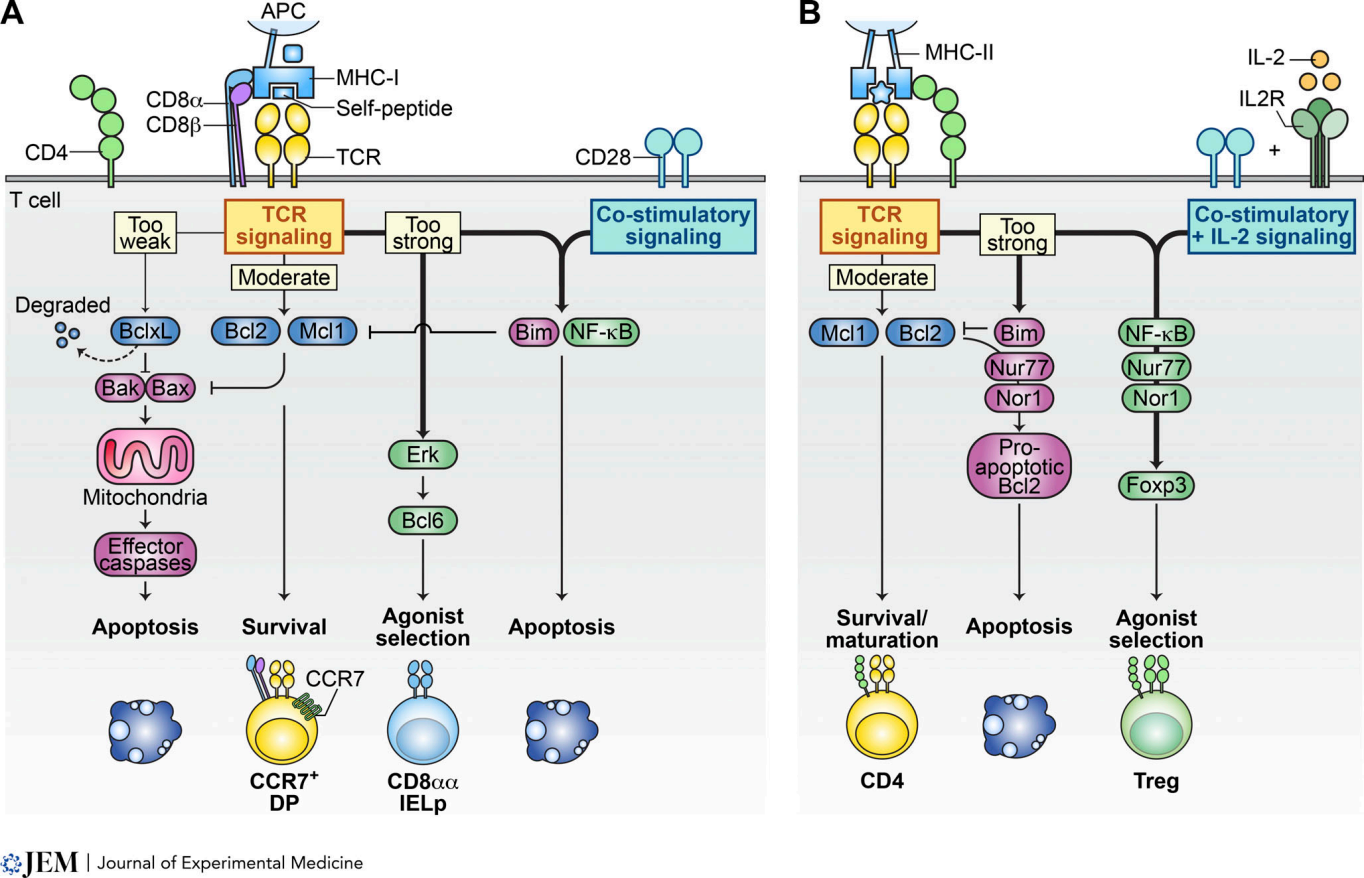
**Differential signaling in agonist selection versus negative selection. (A and B)** This illustration delineates the differential signaling pathways that guide thymocytes undergoing selection thymocytes toward apoptosis or toward agonist selection into CD8αα IEL precursors (IELp; A) or Treg cells (B). **(A)** MHC-I–restricted DP thymocytes receiving moderate TCR signaling can upregulate pro-survival proteins Bcl2 and Mcl1, which take over the survival signaling role from the degrading BclxL. Thymocytes that fail to sufficiently engage TCR signaling to upregulate Bcl2 or Mcl1 will succumb to apoptosis due to BclxL degradation. Conversely, excessively strong TCR signaling leads to thymocyte apoptosis through a mechanism involving NF-κB and pro-apoptotic Bim. Intriguingly, in the absence of NF-κB involvement, MHC-I–restricted thymocytes receiving strong signals may differentiate into CD8αα IEL precursors. This process is facilitated by strong Erk signaling and the upregulation of transcription factor Bcl6, marking an alternative agonist-selection pathway exclusive to MHC-I–restricted thymocytes in the cortex. **(B)** Conversely, MHC-II–restricted thymocytes have an alternative agonist selection pathway leading to Treg cell development in the medulla. Here, moderate TCR signaling is again pivotal for upregulating pro-survival Bcl2 and Mcl1, aiding in the maturation of CD4 T cells. However, overly strong signaling triggers apoptosis. Unlike for CD8αα IEL precursors, co-stimulatory signaling and IL-2 signaling in this context do not induce apoptosis but rather support development of Treg cells.

For thymocytes that do not generate productive TCR signals, their fate hinges on a balance between proapoptotic and antiapoptotic proteins, primarily via the caspase-dependent, Bcl2-regulated pathway (Daley et al., 2017) (Fig. 3). The majority of thymocyte apoptosis occurs in the cortex (Surh and Sprent, 1994), influenced by a sequence of apoptotic regulators (Fig. 3 A). The initial key regulator is the prosurvival protein BclxL, which, upon upregulation by RORγ and RORγt during the transition from CD4^−^CD8^−^ double-negative (DN) to DP thymocytes, starts a 3-day timer for preselection DP thymocytes (Guo et al., 2002; Kurebayashi et al., 2000; Ma et al., 1995; Sun et al., 2000). Within this timeframe, thymocytes must acquire enough positively selecting signals to advance through the thymic selection checkpoints. Failure to do so results in cell death by neglect (Egerton et al., 1990); however, if this period is extended, DP thymocytes may utilize more distal Jα clusters for CDR3 rearrangement, which directly affects the formation of the TCR repertoire (Guo et al., 2002). During this period, weak positive selection signals may induce the upregulation of other pro-survival proteins (e.g., Bcl2 and Mcl1) to substitute for BclxL in counteracting Bim-triggered caspase 3 activation (Alam et al., 1997; Bouillet et al., 1999, 2002; Daley et al., 2017).

### Transmission and enhancement of TCR signals

The 10–20% of preselection thymocytes capable of generating TCR signals, known as signaled DP thymocytes, face the rigorous processes of positive and negative selection and the possibility of being diverted into regulatory and innate-like sublineages. These fate choices are mediated by the TCR signaling machinery, which possesses the capacity to discern subtle variations in self-pMHC ligand interactions, thereby guiding diverse cellular destinies. Engagement of self-pMHC by thymocytes recruits the CD4 or CD8 co-receptor to the TCR complex (Chakraborty and Weiss, 2014; Stepanek et al., 2014; Van Laethem et al., 2013) and triggers the activation of two major non-receptor tyrosine kinases: Lck and Zap70. One feature that can ensure TCR ligand discrimination capacity is the strict hierarchy of the sequential activation of Lck and Zap70. Lck is activated first, initiating the signaling process by phosphorylating the tyrosine residues in CD3 and the ζ-chain. This modification creates docking sites for the kinase Zap70, thereby releasing Zap70 from its autoinhibitory state. Once activated, Zap70 targets two scaffold proteins, LAT and SLP76, which subsequently branch out and amplify TCR signaling via downstream events including the activation of the enzyme PLCγ1 and the Ras-activating protein SOS. This process is highly coordinated: Lck is specialized for the activation of Zap70 and incapable of directly phosphorylating LAT and SLP76. On the other hand, Zap70 is adept at phosphorylating LAT and SLP76, but it cannot phosphorylate immunoreceptor tyrosine-based activation motifs or autoactivate itself. This sequential kinase signaling downstream of the TCR—along with the use of analog rheostats, to modulate incremental gradations of pathway usage, and digital switches, which activate specific signaling pathways only when stimulation exceeds a theshold—provides potential signaling checkpoints to fine-tune and calibrate sensitivity to self-pMHC molecules (Chakraborty and Weiss, 2014; Huseby and Teixeiro, 2022).

**Figure 4.**
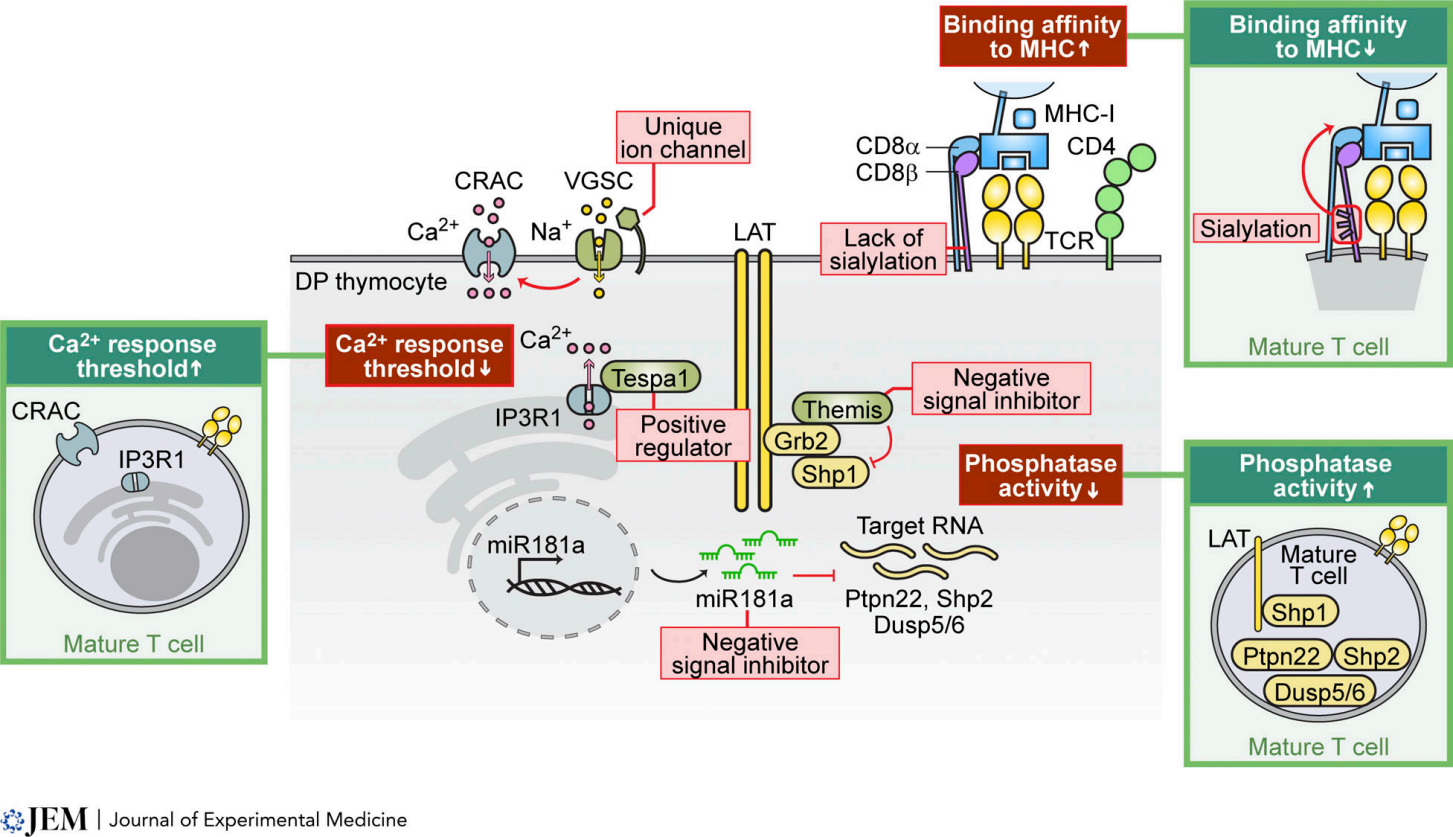
**Regulatory mechanisms for enhanced sensitivity in preselection DP thymocytes.** This illustration depicts the three key molecular mechanisms that confer augmented sensitivity in immature DP thymocytes, enabling their activation by weak positively selecting signals that normally cannot activate mature T cells. First, the sialylation pattern on CD8β determines its binding strength to MHC-I molecules, with the lack of sialylation in DP thymocytes increasing their binding affinity. Second, immature DP thymocytes express stage-specific regulatory proteins that lower response thresholds, augmenting calcium influx. For example, the expression of voltage-gated sodium channels (VGSC) promotes sustained calcium responses through calcium release-activated channels (CRAC) in response to weak ligand stimulation. Additionally, the regulatory protein Tespa1 directly interacts with IP3R1 to enhance calcium release from the endoplasmic reticulum. Lastly, elevated expression of negative signal inhibitors, such as Themis and miR-181a, inhibits phosphatase activity, allowing immature DP thymocytes to be activated by weaker TCR stimuli. The illustrations in the green frames highlight the differential mechanisms in mature T cells, emphasizing the unique features of immature DP thymocytes in the middle.

Various molecular signaling mechanisms have been identified that elucidate how DP thymocytes enhance their ability to use very-weak-affinity self-pMHC interactions to drive cellular differentiation (Fig. 4). The expression of the voltage-gated sodium channel SCN5a–SCN4b and LAT signalosome regulator Tespa1 in DP thymocytes aids intracellular calcium flux following weak self-pMHC interactions (Liang et al., 2017; Lo et al., 2012; Lutes et al., 2021). Meanwhile, Themis expression inhibits the phosphatase activity of Shp-1 (Choi et al., 2023a, 2023b). Additionally, the binding affinity of the CD8αβ co-receptor for MHC molecules is increased in DP thymocytes owing to the lack of specific sialylation patterns (Daniels et al., 2001; Moody et al., 2001). Further, DP thymocytes, unlike their mature counterparts, form a decentralized synapse with multiple, smaller foci accumulating MHC molecules and they do not exhibit actin-mediated TCR movement (Hailman et al., 2002). These unique membrane behaviors allow DP thymocytes to sustain tyrosine phosphorylation events at the APC interface longer than mature T cells, and to migrate effectively from the cortex to the medulla by following chemokine gradients. Reciprocally, en bloc transcriptional changes occur following thymocyte maturation mediated by the downregulation of the microRNA miR181a (Ebert et al., 2009; Li et al., 2007), including the increased expression of the phosphatase CD45, which dephosphorylate TCR complex signaling proteins, inhibitory CD5 signalosomes, and the E3 ligase Cbl-b, which targets signaling molecules for degradation (Azzam et al., 1998; McNeill et al., 2007; Naramura et al., 1998; Tarakhovsky et al., 1995).

In summary, TCR signaling during thymic selection is deemed productive when a thymocyte generates an adequate level of signaling to trigger essential gene regulation for advancement through the developmental stages yet remains moderate enough to not induce apoptosis. A recent study that utilizes single-cell RNA sequencing and single-cell assay for transposase-accessible chromatin with sequencing to delineate signaling attributes across developmental stages provides high-definition insights into the productive signaling traits that are characteristic of each developmental phase (Chopp et al., 2020).

## Thymocyte advancement and lineage branching

**Figure 5.**
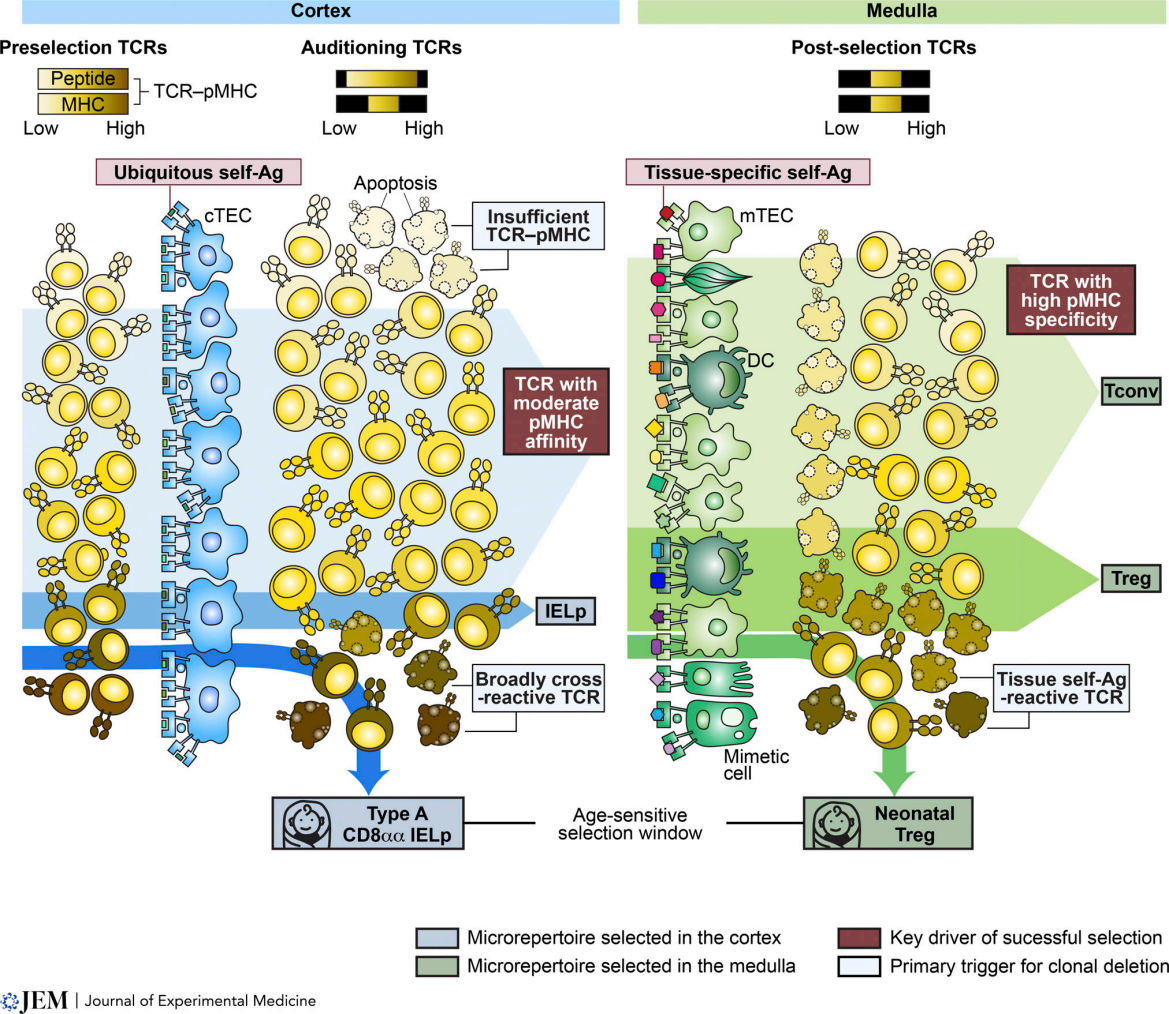
**TCR microrepertoires emerge after thymic checkpoints.** This illustration depicts the formation of TCR microrepertoires emerging after thymic selection in the cortex (left) and medulla (right). Initially, preselection TCRs exhibit a broad range of reactivity toward peptide and MHC molecules. The first stage of selection is mediated by cortical thymic epithelial cells (cTECs), which present ubiquitous self-antigens (Ag). The varying shades of blue represent the strength of TCR–pMHC binding. Following selection, TCRs that bind self-pMHC either too weakly (indicated by white shades) or too strongly are eliminated owing to insufficient TCR–pMHC binding or excessive cross-reactivity. Only TCRs with moderate pMHC binding advance to the medulla for further checkpoint screening (indicated by light blue shades). Sky blue shades denote the TCR signaling strength that guides thymocytes to become IEL precursors (IELp). Notably, a distinct subset of CD8αα IEL precursors (type A) emerges, characterized by an age-dependent selection that is predominant during neonatal stages (represented by the baby icon). In addition, although not shown in the figure, hematopoietic antigen-presenting cells are also key mediators of deletion and CD8αα IEL precursor generation at this checkpoint. In the medulla, remaining thymocytes are screened against tissue-specific antigens, often presented by mTECs (light green cells), dendritic cells (DCs; dark green cells), or mimetic cells (moderate green cells). Mimetic cells mimic tissues through the expression of tissue-specific transcription factors. Examples include muscle mimetic cells (second from the top), ciliated mimetic cells (second from the bottom), or secretory/neuroendocrine mimetic cells (at the bottom). The shades of green indicate the TCR signaling strength during interaction with pMHC. Moderate binding facilitates the development of Tconv cells (light green path), while strong binding leads to the development of Treg cells (moderate green path). Thymocytes experiencing even stronger TCR signaling can differentiate into neonatal Treg cells (the dark green path), a process also bound by a specific temporal selection window limited to a narrow age range in the host (represented by the baby icon).

Regardless of MHC class restriction, signaled DP thymocytes embark on a common trajectory characterized by the increased expression of essential signaling proteins, including TCR, CD3, Zap70, CD69, and CD5. However, within this group of signaled DP thymocytes, the selection mechanisms for MHC-I– and MHC-II–restricted thymocytes start to diverge from the moment DP thymocytes encounter self-pMHC molecules. For MHC-II–restricted thymocytes, indicators of a CD4 T cell gene signature emerge in DP thymocytes before the downregulation of the *Cd8a* and *Cd8b* genes. In contrast, gene signatures indicative of the CD8 T cell lineage emerge during the early CD8 single-positive (CD8SP) stage (Chopp et al., 2020; Steier et al., 2023). Intriguingly, exchanging the gene locations for *Cd4* or *Cd8*, while keeping their cis-regulatory elements intact, does not affect lineage dedication or MHC recognition specificity despite the reversed expression of the CD8 and CD4 proteins. This observation indicates that the level and nature of co-receptor signaling cannot surpass the importance of the divergent transcriptional landscapes in directing the choice between Tconv CD4 and CD8 co-receptors (Shinzawa et al., 2022). It does, however, raise the question of whether the transcriptional divergence of DP signaled thymocytes influences other aspects of thymocyte differentiation, such as the diversion CD8αα IELp. This selection step leads to three principal outcomes: (1) conventional positive selection, wherein thymocytes elevate CCR7 expression, facilitating their entry into the medulla where they undergo co-receptor lineage commitment and continue with further maturation (Takahama, 2006; Lancaster et al., 2018; Baldwin and Robey, 2024; Steier et al., 2024); (2) rerouting into innate-like T cell populations, including MHC-I–restricted CD8αα IEL precursors; or (3) triggering of TCR-mediated apoptosis (stage 1 negative selection) (Fig. 5).

### Fate of DPs with strong self-reactivity: Diversion

When exposed to strong “agonist-selecting” TCR signals, MHC-I– and MHC-II–restricted DP thymocytes have distinct destinies. For MHC-I–restricted DP thymocytes, in addition to negative selection, two unique pathways exist: progression into CD8αα IEL precursors (Gangadharan et al., 2006; Rocha et al., 1991; Yamagata et al., 2004) or exclusion from the thymus via clonal eviction (Badr et al., 2023)—the full significance and limitations of which remain to be fully elucidated. The most self-reactive CD8αα IELs develop within the neonate, a stage of development at which apoptosis is less pronounced (Cheroutre and Lambolez, 2008; Finkel et al., 1992; Huseby et al., 2001). Therefore, there may be an age-dependent switch in the development of MHC-I–signaled DP thymocytes, shifting from innate-like cell development (favored during neonatal stages) toward clonal eviction and/or apoptosis (favored in adult stages) (Fig. 5). Conversely, MHC-II–restricted DP thymocytes generally do not follow the pathway leading to the IEL precursor state nor do they undergo clonal eviction. However, similar “agonist-selecting” TCR signal intensities encountered at the CD4SP stage can lead MHC-II–restricted cells to either negative selection or differentiation into Treg cells, a route less accessible to their MHC-I–restricted counterparts (Fig. 2). CD8αα IEL precursor commitment in the thymic cortex is marked by their high expression of TCR and PD-1—indicators of recent TCR signaling activation—and the absence of CCR7, which usually guides thymocytes to the medulla. This suggests that CD8αα IEL precursors, unlike Treg cells, are selected in the cortex (Ruscher et al., 2017). These findings, alongside related studies, highlight that thymocytes with high affinity for self-ligands are either deleted or differentiate into specific agonist-selected lineages: Treg cells for MHC-II–restricted thymocytes and CD8αα IEL precursors for MHC-I–restricted ones.

Understanding how CD8αα IEL precursor development coordinates with the usual patterns of co-receptor expression has garnered significant interest (Issuree et al., 2017). Investigating this relationship could provide insights into why MHC-II–restricted thymocytes seldom choose this developmental path. It is well established that one significant phenotypic change in immature DP thymocytes upon receiving selecting TCR signals is the transition to a “dulled” state (Kersh and Hedrick, 1995; McGargill and Hogquist, 1999), characterized by the downregulation of both CD4 and CD8 co-receptors. These TCR-signaled DP thymocytes are yet to commit to a specific lineage and to accurately determine their MHC recognition specificity and co-receptor selection. The kinetic signaling model posits that DP thymocytes initially cease *Cd8* gene transcription and then assess the impact of CD8’s absence on TCR signaling (Singer et al., 2008). If TCR-mediated positive selection signals persist without *Cd8* transcription, thymocytes mature into CD4 T cells. If, however, TCR-mediated positive selection signals cease in the absence of *Cd8* transcription, the thymocytes develop into CD8 T cells. This framework, which categorizes signaling as either continued or halted, underscores the significance of stage-specific regulation of CD8 expression through cis-regulatory elements in determining thymic selection outcomes.

On a molecular level, at the immature DP thymocyte stage, CD8 expression is primarily driven by enhancers E8_II_ and E8_III_. In contrast, enhancer E8_IV_ is responsible for driving CD8αα expression on CD8SP thymocytes and mature T cells, whereas the constitutive expression of CD8αα on IELs is regulated by enhancer E8_I_ (Cheroutre and Lambolez, 2008). The unique usage of CD8αα, instead of CD8αβ, as a co-receptor may contribute to the survival and function of highly self-reactive clones as it dampens TCR signaling by redirecting key signaling molecules, such as Lck, away from the TCR, consequently diminishing the sensitivity of the TCR to pMHC (Cheroutre and Lambolez, 2008). The differential use of various cis-regulatory elements to coordinate CD8α and CD8β expression enables distinct transcription factors to regulate the expression of these co-receptors at specific developmental stages (Issuree et al., 2017). In addition, these MHC-I–restricted dulled DP thymocytes, when subjected to agonist-selecting signals, may tip the balance from apoptosis toward survival through the Ras activator RasGRP1 and the transcription factor Bcl6 (Golec et al., 2017; Xing et al., 2024). Specifically, Bcl6 expression is upregulated by TCR signaling, particularly through the Erk pathway (Xing et al., 2024), facilitating the maturation of innate-like thymocytes during agonist selection.

In sharp contrast, DP thymocytes restricted to MHC-II are generally not incorporated into the IEL lineage. Weak TCR signals, derived from “non-cognate” self-pMHC-II ligands, interactions too weak to induce mature CD4 T cell proliferation, can nonetheless initiate negative selection in vivo (Sant’Angelo and Janeway, 2002; Stadinski et al., 2023). This suggests that the threshold for negative selection may be differentially regulated for ligands bound to MHC-I versus MHC-II molecules. Indeed, studies examining the survival rates of autoreactive thymocytes post-negative selection have indicated that in response to a comparable level of TCR stimulation—enough to induce upregulation of the activation marker PD-1 and expression of TCRβ—a significantly higher frequency of MHC-I–specific autoreactive thymocytes manages to evade negative selection compared with their MHC-II–specific counterparts (Badr et al., 2023). This observation then raises the question of whether the initial discrepancies observed in cortical thymocytes signaled by MHC-I versus MHC-II result from direct interactions between TCR–pMHC complexes or if they are due to variations in the strength of MHC binding and the signals transmitted through the co-receptors (Cheroutre and Lambolez, 2008; Moody et al., 2001; Stepanek et al., 2014). A deeper investigation is needed to understand how these discrepancies influence the thymocyte decision-making process in the cortex, and whether a parallel lineage to CD8αα IEL precursors exists for MHC-II–restricted, signaled DP thymocytes. The detailed interactions between TCR, pMHC, and co-receptors, which shape the journey of DP thymocytes, underscore the elaborate molecular blueprint that assembles each TCR microrepertoire. Unraveling this complexity demands comprehensive research to decode the specific functions and influences of each component involved in thymic selection. It is also imperative to examine if early signaling experiences in the cortex imprint functional specializations on mature TCR microrepertoires, potentially affecting the broader dynamics of immune responses and tolerance.

### Fate of DPs with strong self-reactivity: Death

The precise mechanisms through which thymocytes undergo TCR-induced clonal elimination, and whether recognition of cognate and non-cognate self-pMHC activate the same cell death pathways during negative selection, remain active areas of study. The proapoptotic effects of Bim and Puma are gene dose dependent (Bouillet et al., 1999; Gray et al., 2012) and correlate with the strength of TCR signaling (Hojo et al., 2019), mirroring the induction of Nur77—a protein considered crucial for central tolerance (Hiwa et al., 2021, 2022). Both proteins, while not strictly necessary for negative selection in the cortex, are pivotal for stage 2 negative selection in the medulla (Fig. 3 B) (Hiwa et al., 2021; Hu and Baldwin, 2015). Bim deficiency can salvage the Nur77^hi^ population (Stritesky et al., 2013), and evidence indicates Bim could be regulated by Nur77 at the transcriptional level (Bouillet et al., 2002; Fassett et al., 2012), suggesting a possible dynamic interplay between the two. Interestingly, Nur77 can also move to the mitochondria to trigger apoptosis in a Bim/caspase-independent manner, converting Bcl2 into a proapoptotic agent (Fig. 3 B) (Fassett et al., 2012; Rajpal et al., 2003; Thompson and Winoto, 2008; Wang et al., 2009)—a process significantly hampered by TCR signaling activation during the selection process (Hu and Baldwin, 2015). This observation suggests a molecular dialogue in which Nur77 helps decide between clonal deletion and tolerance induction. A key hint may be gleaned from a study that explored the impact of Nur77 and Nor1 deficiency in DP thymocytes through competitive bone marrow chimeras. This study revealed that even the presence of Treg cells restored by wild-type bone marrow fails to halt autoimmune tendencies in T cells devoid of both Nur77 and Nor1 (Hiwa et al., 2021). This implies that Nur77 plays a critical role beyond clonal deletion and Treg development, specifically in initiating signaling adaptations that mitigate self-reactivity in T cells escaping negative selection. The roles of cytokines and glucocorticoids, and their influence on thymocyte signaling will need to be incorporated into our overall understanding of the death pathway activation (Burger et al., 2014; McCarron et al., 2019; Taves and Ashwell, 2021).

### Medullary thymocyte branching

Following the initial stages of positive selection, thymocytes that are neither diverted nor deleted begin to downregulate the CD8 co-receptor and commit to either the CD4 or CD8 lineage, followed by migration to the thymic medulla (Steier et al., 2024). During their journey through the thymus, thymocytes encounter self-pMHC molecules presented by a network of thymic dendritic cells at the cortico-medullary junction and by medullary thymic epithelial cells (mTECs) at various stages of their differentiation (Fig. 5). These interactions between thymocytes and APCs in the thymic medulla initiate a second wave of selection (Figs. 2 and 5). This phase allows thymocytes to either progress in the Tconv differentiation pathway or be eliminated through negative selection. For CD4SP thymocytes, and to a lesser extent CD8SP thymocytes, interactions with self-pMHC complexes of moderately high affinity can trigger a rescue from apoptosis and redirection into the Treg cell lineage (Figs. 2 and 5). This rescue is facilitated when NF-κB–mediated signaling counteracts the proapoptotic function of Bim, thereby protecting developing thymocytes from being deleted (Li and Rudensky, 2016; Klein et al., 2019; Baldwin and Robey, 2024) (Fig. 3 B). Cytokine signaling, especially via IL-2, can further inhibit proapoptotic signals, supporting the development of Treg cells (Hemmers et al., 2019; Hu et al., 2017; Klein et al., 2019) (Fig. 3 B). After this second selection phase, thymocytes complete their maturation and join the mature T cell pool. Recent reviews have extensively covered the diversion of thymocytes into Treg cells in the thymus (Dikiy and Rudensky, 2023; Sakaguchi et al., 2020; Savage et al., 2020); therefore, we will focus on how the developmental stage of thymocyte selection or diversion and the presentation of self-antigens influence the specificities of the TCRs expressed on different T cell subsets.

## The specificities of TCRs expressed on T cell subsets

Why are thymocytes subject to two stages of negative selection and diversion? One potential answer to this question lies in the inherent difference in the quality of the self-antigens present in the cortex as compared with those in the medulla; each region is characterized by semi-distinct immunopeptidomes created via the expression of unique, region-specific proteosomes and proteases (Klein et al., 2019; Murata et al., 2007). The exclusive expression of Aire—a transcription factor critical for the regulation of tissue-specific antigen expression—in mTEC signifies the compartmentalization of distinct immunopeptidomes in the cortex versus the medulla (Mathis and Benoist, 2009). Recent studies indicate that apart from Aire inducing tissue-specific gene expression by repurposing general transcriptional mechanisms, subsets of Aire^+^ mTECs may also express lineage-defining transcription factors that allow for their further differentiation into “mimetic cells”—mTECs that express transcription factors and carry tissue-specific chromatin landscapes, which mimic peripheral cells and allow for tissue-specific antigen expression and presentation within the medulla (Farr and Rudensky, 1998; Michelson et al., 2022, 2023; Sin et al., 2023). Thus, within the thymic realm, the thymic cortex and medulla present markedly disparate immunopeptidomes: while the cortex showcases a repertoire of ubiquitous self-antigens, the medulla serves as a theater for the exhibition of tissue-specific counterparts. These distinct self-antigen profiles orchestrate a nuanced journey for developing thymocytes, imbuing them with distinct signaling capacities (Fig. 5).

One distinguishing factor between the early/cortical and late/medullary stages of thymocyte diversion and negative selection is the primary objective of the former to eliminate Tconv cells from expressing TCR clonotypes that display extensive affinity for or cross-reactivity to pMHC ligands (Fig. 5). The initial branching may also be biased toward the development of neonatal-derived innate-like T cells with TCRs that are broadly self-reactive and thus focused on reading out changes in the expression levels of MHC molecules rather than the presence or absence of a foreign ligand (Cheroutre et al., 2011). This initial stage sets the foundation for the latter stage to focus on screening TCR clones based on their capacity to recognize particular self-peptide antigens (Fig. 5). In essence, the two stages of negative selection may pertain to the two anatomical settings of negative selection and T cell diversion. Yet, the outstanding question is whether these stages have unique functions in the formation of TCR microrepertoires.

One approach to test this theoretical framework is to scrutinize the resultant TCR microrepertoires, examining the clones that are eliminated or persist through each phase of thymic selection. Notably, when preselection TCR clones were assessed in vivo, DP thymocytes that expressed TCRs reactive to multiple self-pMHCs underwent negative selection (Huseby et al., 2005; Stadinski et al., 2011), or, when rescued from negative selection, could be diverted into CD8αα IEL precursors (McDonald et al., 2015). This hypothesis has been further validated by characterizing the biochemical properties of TCRs that target thymocytes for negative selection and those of cells that matured into CD8αα IEL precursors (Stadinski et al., 2016; Wirasinha et al., 2018). The lack of CCR7 expression indicated that these cells were receiving negative selection and diversion signals in the thymic cortex (McDonald et al., 2015).

In contrast, self-antigens critical to medullary negative selection and Foxp3^+^ Treg diversion primarily consist of tissue-specific peptides, age-dependent antigens, and self-epitopes that are upregulated by inflammation (Hassler et al., 2019; Kieback et al., 2016; Leonard et al., 2017; Stadinski et al., 2019). Similar to their innate-like T cell counterparts, neonatal Treg cells appear to carry unique, highly self-reactive clones and possess unique anti-inflammatory characteristics (Stadinski et al., 2019; Yang et al., 2015) (Fig. 5). Whether age-dependent alterations in Treg repertoires derive from alterations in antigen presentation, susceptibility to apoptosis, or TCR signaling has not been fully elucidated. However, unlike their poly-pMHC-reactive CD8αα IEL precursor counterparts, Treg cells appear to express TCRs that have high affinity and high specificity for particular self-antigens. Indeed, tetramer staining and surface plasmon resonance studies indicated thymic Foxp3^+^ Treg cells have high affinity for their targets as compared with CD4 Tconv cells that recognize the same antigen and require expression of the target antigen for development (Hassler et al., 2019; Kalekar et al., 2016; Kieback et al., 2016; Legoux et al., 2015; Leonard et al., 2017; Stadinski et al., 2019). Exquisite Treg cell specificity likely endows individual clones with the ability to function in a spatially segregated, cell-autonomous manner during health and disease (Kaminski et al., 2023; Muñoz-Rojas and Mathis, 2021).

## Final thoughts and key takeaways

The publication of Burnet’s groundbreaking “The Clonal Selection Theory of Acquired Immunity,” subsequent to his Nobel Prize recognition, firmly established the critical role of immune tolerance in immunology. The foundational status of self-tolerance, both within the thymus and in the periphery, is widely accepted even as our understanding of the principles continued to be refined by ongoing research. The concept of clonal diversion highlights the functional specialization of T cell microrepertoires and the crucial role of “thymic imprinting” as a potential basis for such specialization. Anatomically, the thymus is divided into two essential areas—the cortex and the medulla. It is well documented that thymocyte development and maturation proceed along a predetermined path, involving interactions with specialized epithelial cells and APCs to fulfill developmental milestones. This review aims to integrate recent advancements in the field into a broader framework to demonstrate how the development of each individual microrepertoire and the balance between selection and elimination are intimately connected with the migratory pathways of thymocytes. A core element of T cell selection is the differing quality of selecting ligands in the cortex and medulla, which in turn influences the quality of post-selection TCRs. These distinctive ligand panels create TCR microrepertoires at the different anatomical locations within the thymus, better equipping various sublineages of T cells—be they Tconv, Treg, or innate-like cells—for their specific functional roles.

Drawing on a cultural reference, the ancient Chinese tale of “Mencius’s mother, three moves” serves as an illustrative analogy. This story, which recounts how the mother of famed Confucian philosopher Mencius moved their home three times to secure a conducive environment for her son’s education, stresses the importance of setting for developmental growth. Similarly, during thymic selection, thymocytes undergo several “moves,” resulting in the selection of TCRs belonging to three major groups along the way (Fig. 1). In the first move, immature thymocytes temporarily reside in and explore the cortex. During this phase, the presence of ubiquitous self-antigens emphasizes thymic education aimed at reducing overreactivity toward MHC molecules, thereby ensuring self-restriction (Figs. 2 and 5). In the second move, the remaining thymocytes migrate to the medulla, where they undergo education on tissue-specific antigens. This stage involves the presentation of a plethora of tissue-specific antigens via Aire-dependent or mimetic cell–mediated mechanisms (Figs. 2 and 5). This step not only eliminates thymocytes that are overly reactive to self-antigens but also tunes the thymocytes’ reactivity thresholds to prevent reaction with tissue-specific antigens. Importantly, during both educational moves, alternative pathways, apart from cell death, become available, including commitment to CD8αα IEL precursor fate in the cortex and Treg cell fate in the medulla. Although the association of MHC-I versus MHC-II restriction with these anatomically specific alternative lineages remains to be fully understood, these alternative fates demonstrate the fundamental distinction in pMHC recognition between Tconv, Treg cells, and IEL precursors. The selection of T cell microrepertoires highlights the intricate, complex interplay between selection and elimination at the signaling level, while also revealing the elegant allocation and partitioning of TCR microrepertoires for their most effective uses.

## References

[bib1] Alam, A., M.Y. Braun, F. Hartgers, S. Lesage, L. Cohen, P. Hugo, F. Denis, and R.P. Sékaly. 1997. Specific activation of the cysteine protease CPP32 during the negative selection of T cells in the thymus. J. Exp. Med. 186:1503–1512. 10.1084/jem.186.9.1503 9348308 PMC2199117

[bib2] Arstila, T.P., A. Casrouge, V. Baron, J. Even, J. Kanellopoulos, and P. Kourilsky. 1999. A direct estimate of the human alphabeta T cell receptor diversity. Science. 286:958–961. 10.1126/science.286.5441.958 10542151

[bib3] Azzam, H.S., A. Grinberg, K. Lui, H. Shen, E.W. Shores, and P.E. Love. 1998. CD5 expression is developmentally regulated by T cell receptor (TCR) signals and TCR avidity. J. Exp. Med. 188:2301–2311. 10.1084/jem.188.12.2301 9858516 PMC2212429

[bib4] Badr, M.E., Z. Zhang, X. Tai, and A. Singer. 2023. CD8 T cell tolerance results from eviction of immature autoreactive cells from the thymus. Science. 382:534–541. 10.1126/science.adh4124 37917689 PMC11302524

[bib142] Baldwin, I., and E.A. Robey. 2024. Adjusting to self in the thymus: CD4 versus CD8 lineage commitment and regulatory T cell development. J. Exp. Med. 221. 10.1084/jem.20230896 PMC11232887 38980291

[bib5] Berg, L.J., A.M. Pullen, B. Fazekas de St Groth, D. Mathis, C. Benoist, and M.M. Davis. 1989. Antigen/MHC-specific T cells are preferentially exported from the thymus in the presence of their MHC ligand. Cell. 58:1035–1046. 10.1016/0092-8674(89)90502-3 2476238

[bib6] Bevan, M.J. 1977. In a radiation chimaera, host H-2 antigens determine immune responsiveness of donor cytotoxic cells. Nature. 269:417–418. 10.1038/269417a0 302918

[bib7] Billingham, R.E., L. Brent, and P.B. Medawar. 1953. Actively acquired tolerance of foreign cells. Nature. 172:603–606. 10.1038/172603a0 13099277

[bib8] Blackman, M., J. Kappler, and P. Marrack. 1990. The role of the T cell receptor in positive and negative selection of developing T cells. Science. 248:1335–1341. 10.1126/science.1972592 1972592

[bib9] Bouillet, P., D. Metcalf, D.C. Huang, D.M. Tarlinton, T.W. Kay, F. Köntgen, J.M. Adams, and A. Strasser. 1999. Proapoptotic Bcl-2 relative Bim required for certain apoptotic responses, leukocyte homeostasis, and to preclude autoimmunity. Science. 286:1735–1738. 10.1126/science.286.5445.1735 10576740

[bib10] Bouillet, P., J.F. Purton, D.I. Godfrey, L.C. Zhang, L. Coultas, H. Puthalakath, M. Pellegrini, S. Cory, J.M. Adams, and A. Strasser. 2002. BH3-only Bcl-2 family member Bim is required for apoptosis of autoreactive thymocytes. Nature. 415:922–926. 10.1038/415922a 11859372

[bib11] Bradley, P., and P.G. Thomas. 2019. Using T cell receptor repertoires to understand the principles of adaptive immune recognition. Annu. Rev. Immunol. 37:547–570. 10.1146/annurev-immunol-042718-041757 30699000

[bib12] Burger, M.L., K.K. Leung, M.J. Bennett, and A. Winoto. 2014. T cell-specific inhibition of multiple apoptotic pathways blocks negative selection and causes autoimmunity. Elife. 3:e03468. 10.7554/eLife.03468 25182415 PMC4171708

[bib13] Burnet, F.M. 1976. A modification of Jerne’s theory of antibody production using the concept of clonal selection. CA Cancer J. Clin. 26:119–121. 10.3322/canjclin.26.2.119 816431

[bib14] Burnet, F.M. 1961. Immunological recognition of self. Science. 133:307–311. 10.1126/science.133.3449.307 13689158

[bib15] Burnet, F.M. 1991. The Nobel lectures in immunology. The Nobel prize for physiology or medicine, 1960. Scand. J. Immunol. 33:3–13. 1996406 10.1111/j.1365-3083.1991.tb02487.x

[bib16] Chakraborty, A.K., and A. Weiss. 2014. Insights into the initiation of TCR signaling. Nat. Immunol. 15:798–807. 10.1038/ni.2940 25137454 PMC5226627

[bib17] Cheroutre, H., and F. Lambolez. 2008. Doubting the TCR coreceptor function of CD8alphaalpha. Immunity. 28:149–159. 10.1016/j.immuni.2008.01.005 18275828

[bib18] Cheroutre, H., F. Lambolez, and D. Mucida. 2011. The light and dark sides of intestinal intraepithelial lymphocytes. Nat. Rev. Immunol. 11:445–456. 10.1038/nri3007 21681197 PMC3140792

[bib19] Choi, S., T. Hatzihristidis, G. Gaud, A. Dutta, J. Lee, A. Arya, L.M. Clubb, D.B. Stamos, A. Markovics, K. Mikecz, and P.E. Love. 2023a. GRB2 promotes thymocyte positive selection by facilitating THEMIS-mediated inactivation of SHP1. J. Exp. Med. 220:e20221649. 10.1084/jem.20221649 37067793 PMC10114920

[bib20] Choi, S., J. Lee, T. Hatzihristidis, G. Gaud, A. Dutta, A. Arya, L.M. Clubb, D.B. Stamos, A. Markovics, K. Mikecz, and P.E. Love. 2023b. THEMIS increases TCR signaling in CD4^+^CD8^+^ thymocytes by inhibiting the activity of the tyrosine phosphatase SHP1. Sci. Signal. 16:eade1274. 10.1126/scisignal.ade1274 37159521 PMC10410529

[bib21] Chopp, L.B., V. Gopalan, T. Ciucci, A. Ruchinskas, Z. Rae, M. Lagarde, Y. Gao, C. Li, M. Bosticardo, F. Pala, . 2020. An integrated epigenomic and transcriptomic map of mouse and human αβ T cell development. Immunity. 53:1182–1201.e8. 10.1016/j.immuni.2020.10.024 33242395 PMC8641659

[bib22] Cudkowicz, G., and G.B. Rossi. 1972. Hybrid resistance to parental DBA-2 grafts: Independence from the H-2 locus. I. Studies with normal hematopoietic cells. J. Natl. Cancer Inst. 48:131–139. 4405704

[bib23] Dai, S., E.S. Huseby, K. Rubtsova, J. Scott-Browne, F. Crawford, W.A. Macdonald, P. Marrack, and J.W. Kappler. 2008. Crossreactive T Cells spotlight the germline rules for alphabeta T cell-receptor interactions with MHC molecules. Immunity. 28:324–334. 10.1016/j.immuni.2008.01.008 18308592 PMC2287197

[bib24] Daley, S.R., C. Teh, D.Y. Hu, A. Strasser, and D.H.D. Gray. 2017. Cell death and thymic tolerance. Immunol. Rev. 277:9–20. 10.1111/imr.12532 28462532

[bib25] Daniels, M.A., L. Devine, J.D. Miller, J.M. Moser, A.E. Lukacher, J.D. Altman, P. Kavathas, K.A. Hogquist, and S.C. Jameson. 2001. CD8 binding to MHC class I molecules is influenced by T cell maturation and glycosylation. Immunity. 15:1051–1061. 10.1016/S1074-7613(01)00252-7 11754824

[bib26] Davis, M.M. 2015. Not-so-negative selection. Immunity. 43:833–835. 10.1016/j.immuni.2015.11.002 26588773

[bib27] Davis, M.M., and P.J. Bjorkman. 1988. T-cell antigen receptor genes and T-cell recognition. Nature. 334:395–402. 10.1038/334395a0 3043226

[bib28] Dikiy, S., and A.Y. Rudensky. 2023. Principles of regulatory T cell function. Immunity. 56:240–255. 10.1016/j.immuni.2023.01.004 36792571

[bib29] Ebert, P.J., S. Jiang, J. Xie, Q.J. Li, and M.M. Davis. 2009. An endogenous positively selecting peptide enhances mature T cell responses and becomes an autoantigen in the absence of microRNA miR-181a. Nat. Immunol. 10:1162–1169. 10.1038/ni.1797 19801983 PMC3762483

[bib30] Egerton, M., R. Scollay, and K. Shortman. 1990. Kinetics of mature T-cell development in the thymus. Proc. Natl. Acad. Sci. USA. 87:2579–2582. 10.1073/pnas.87.7.2579 2138780 PMC53733

[bib31] Farr, A.G., and A. Rudensky. 1998. Medullary thymic epithelium: A mosaic of epithelial “self”? J. Exp. Med. 188:1–4. 10.1084/jem.188.1.1 9653078 PMC2525576

[bib32] Fassett, M.S., W. Jiang, A.M. D’Alise, D. Mathis, and C. Benoist. 2012. Nuclear receptor Nr4a1 modulates both regulatory T-cell (Treg) differentiation and clonal deletion. Proc. Natl. Acad. Sci. USA. 109:3891–3896. 10.1073/pnas.1200090109 22345564 PMC3309794

[bib33] Finkel, T.H., J.W. Kappler, and P.C. Marrack. 1992. Immature thymocytes are protected from deletion early in ontogeny. Proc. Natl. Acad. Sci. USA. 89:3372–3374. 10.1073/pnas.89.8.3372 1565628 PMC48869

[bib34] Gangadharan, D., F. Lambolez, A. Attinger, Y. Wang-Zhu, B.A. Sullivan, and H. Cheroutre. 2006. Identification of pre- and postselection TCRalphabeta+ intraepithelial lymphocyte precursors in the thymus. Immunity. 25:631–641. 10.1016/j.immuni.2006.08.018 17045820

[bib35] Garcia, K.C., M. Degano, R.L. Stanfield, A. Brunmark, M.R. Jackson, P.A. Peterson, L. Teyton, and I.A. Wilson. 1996. An alphabeta T cell receptor structure at 2.5 A and its orientation in the TCR-MHC complex. Science. 274:209–219. 10.1126/science.274.5285.209 8824178

[bib36] Garman, R.D., J.L. Ko, C.D. Vulpe, and D.H. Raulet. 1986. T-cell receptor variable region gene usage in T-cell populations. Proc. Natl. Acad. Sci. USA. 83:3987–3991. 10.1073/pnas.83.11.3987 3487085 PMC323650

[bib37] Golec, D.P., R.E. Hoeppli, L.M. Henao Caviedes, J. McCann, M.K. Levings, and T.A. Baldwin. 2017. Thymic progenitors of TCRαβ^+^ CD8αα intestinal intraepithelial lymphocytes require RasGRP1 for development. J. Exp. Med. 214:2421–2435. 10.1084/jem.20170844 28652304 PMC5551581

[bib38] Gray, D.H., F. Kupresanin, S.P. Berzins, M.J. Herold, L.A. O’Reilly, P. Bouillet, and A. Strasser. 2012. The BH3-only proteins Bim and Puma cooperate to impose deletional tolerance of organ-specific antigens. Immunity. 37:451–462. 10.1016/j.immuni.2012.05.030 22960223 PMC3500635

[bib39] Guo, J., A. Hawwari, H. Li, Z. Sun, S.K. Mahanta, D.R. Littman, M.S. Krangel, and Y.W. He. 2002. Regulation of the TCRalpha repertoire by the survival window of CD4(+)CD8(+) thymocytes. Nat. Immunol. 3:469–476. 10.1038/ni791 11967541

[bib40] Hailman, E., W.R. Burack, A.S. Shaw, M.L. Dustin, and P.M. Allen. 2002. Immature CD4(+)CD8(+) thymocytes form a multifocal immunological synapse with sustained tyrosine phosphorylation. Immunity. 16:839–848. 10.1016/S1074-7613(02)00326-6 12121665

[bib41] Hassler, T., E. Urmann, S. Teschner, C. Federle, T. Dileepan, K. Schober, M.K. Jenkins, D.H. Busch, M. Hinterberger, and L. Klein. 2019. Inventories of naive and tolerant mouse CD4 T cell repertoires reveal a hierarchy of deleted and diverted T cell receptors. Proc. Natl. Acad. Sci. USA. 116:18537–18543. 10.1073/pnas.1907615116 31451631 PMC6744931

[bib42] Hemmers, S., M. Schizas, E. Azizi, S. Dikiy, Y. Zhong, Y. Feng, G. Altan-Bonnet, and A.Y. Rudensky. 2019. IL-2 production by self-reactive CD4 thymocytes scales regulatory T cell generation in the thymus. J. Exp. Med. 216:2466–2478. 10.1084/jem.20190993 31434685 PMC6829602

[bib43] Hiwa, R., J.F. Brooks, J.L. Mueller, H.V. Nielsen, and J. Zikherman. 2022. NR4A nuclear receptors in T and B lymphocytes: Gatekeepers of immune tolerance. Immunol. Rev. 307:116–133. 10.1111/imr.13072 35174510

[bib44] Hiwa, R., H.V. Nielsen, J.L. Mueller, R. Mandla, and J. Zikherman. 2021. NR4A family members regulate T cell tolerance to preserve immune homeostasis and suppress autoimmunity. JCI Insight. 6:e151005. 10.1172/jci.insight.151005 34343134 PMC8492309

[bib45] Hogquist, K.A., and S.C. Jameson. 2014. The self-obsession of T cells: How TCR signaling thresholds affect fate “decisions” and effector function. Nat. Immunol. 15:815–823. 10.1038/ni.2938 25137456 PMC4348363

[bib46] Hogquist, K.A., S.C. Jameson, W.R. Heath, J.L. Howard, M.J. Bevan, and F.R. Carbone. 1994. T cell receptor antagonist peptides induce positive selection. Cell. 76:17–27. 10.1016/0092-8674(94)90169-4 8287475

[bib47] Hojo, M.A., K. Masuda, H. Hojo, Y. Nagahata, K. Yasuda, D. Ohara, Y. Takeuchi, K. Hirota, Y. Suzuki, H. Kawamoto, and S. Kawaoka. 2019. Identification of a genomic enhancer that enforces proper apoptosis induction in thymic negative selection. Nat. Commun. 10:2603. 10.1038/s41467-019-10525-1 31197149 PMC6565714

[bib48] Hosokawa, H., and E.V. Rothenberg. 2020. How transcription factors drive choice of the T cell fate. Nat. Rev. Immunol. 21:162–176. 10.1038/s41577-020-00426-6 32918063 PMC7933071

[bib49] Hu, D.Y., R.C. Wirasinha, C.C. Goodnow, and S.R. Daley. 2017. IL-2 prevents deletion of developing T-regulatory cells in the thymus. Cell Death Differ. 24:1007–1016. 10.1038/cdd.2017.38 28362433 PMC5442470

[bib50] Hu, Q.N., and T.A. Baldwin. 2015. Differential roles for Bim and Nur77 in thymocyte clonal deletion induced by ubiquitous self-antigen. J. Immunol. 194:2643–2653. 10.4049/jimmunol.1400030 25687757

[bib51] Huseby, E., J. Kappler, and P. Marrack. 2004. TCR-MHC/peptide interactions: Kissing-cousins or a shotgun wedding? Eur. J. Immunol. 34:1243–1250. 10.1002/eji.200425000 15114657

[bib52] Huseby, E.S., B. Sather, P.G. Huseby, and J. Goverman. 2001. Age-dependent T cell tolerance and autoimmunity to myelin basic protein. Immunity. 14:471–481. 10.1016/S1074-7613(01)00127-3 11336692

[bib53] Huseby, E.S., and E. Teixeiro. 2022. The perception and response of T cells to a changing environment are based on the law of initial value. Sci. Signal. 15:eabj9842. 10.1126/scisignal.abj9842 35639856 PMC9290192

[bib54] Huseby, E.S., J. White, F. Crawford, T. Vass, D. Becker, C. Pinilla, P. Marrack, and J.W. Kappler. 2005. How the T cell repertoire becomes peptide and MHC specific. Cell. 122:247–260. 10.1016/j.cell.2005.05.013 16051149

[bib55] Issuree, P.D., C.P. Ng, and D.R. Littman. 2017. Heritable gene regulation in the CD4:CD8 T cell lineage choice. Front. Immunol. 8:291. 10.3389/fimmu.2017.00291 28382035 PMC5360760

[bib56] Itano, A., and E. Robey. 2000. Highly efficient selection of CD4 and CD8 lineage thymocytes supports an instructive model of lineage commitment. Immunity. 12:383–389. 10.1016/S1074-7613(00)80190-9 10795736

[bib57] Jerne, N.K. 1971. The somatic generation of immune recognition. Eur. J. Immunol. 1:1–9. 10.1002/eji.1830010102 14978855

[bib58] Josefowicz, S.Z., L.F. Lu, and A.Y. Rudensky. 2012. Regulatory T cells: Mechanisms of differentiation and function. Annu. Rev. Immunol. 30:531–564. 10.1146/annurev.immunol.25.022106.141623 22224781 PMC6066374

[bib59] Kalekar, L.A., S.E. Schmiel, S.L. Nandiwada, W.Y. Lam, L.O. Barsness, N. Zhang, G.L. Stritesky, D. Malhotra, K.E. Pauken, J.L. Linehan, . 2016. CD4(+) T cell anergy prevents autoimmunity and generates regulatory T cell precursors. Nat. Immunol. 17:304–314. 10.1038/ni.3331 26829766 PMC4755884

[bib60] Kaminski, A., F.T. Hager, L. Kopplin, F. Ticconi, A. Leufgen, E. Vendelova, L. Rüttger, G. Gasteiger, V. Cerovic, W. Kastenmüller, . 2023. Resident regulatory T cells reflect the immune history of individual lymph nodes. Sci. Immunol. 8:eadj5789. 10.1126/sciimmunol.adj5789 37874251

[bib61] Kappler, J.W., N. Roehm, and P. Marrack. 1987. T cell tolerance by clonal elimination in the thymus. Cell. 49:273–280. 10.1016/0092-8674(87)90568-X 3494522

[bib62] Kersh, G.J., and S.M. Hedrick. 1995. Role of TCR specificity in CD4 versus CD8 lineage commitment. J. Immunol. 154:1057–1068. 10.4049/jimmunol.154.3.1057 7822783

[bib63] Kieback, E., E. Hilgenberg, U. Stervbo, V. Lampropoulou, P. Shen, M. Bunse, Y. Jaimes, P. Boudinot, A. Radbruch, U. Klemm, . 2016. Thymus-derived regulatory T cells are positively selected on natural self-antigen through cognate interactions of high functional avidity. Immunity. 44:1114–1126. 10.1016/j.immuni.2016.04.018 27192577

[bib64] Kisielow, P., H. Blüthmann, U.D. Staerz, M. Steinmetz, and H. von Boehmer. 1988. Tolerance in T-cell-receptor transgenic mice involves deletion of nonmature CD4+8+ thymocytes. Nature. 333:742–746. 10.1038/333742a0 3260350

[bib65] Klein, L., E.A. Robey, and C.S. Hsieh. 2019. Central CD4^+^ T cell tolerance: Deletion versus regulatory T cell differentiation. Nat. Rev. Immunol. 19:7–18. 10.1038/s41577-018-0083-6 30420705

[bib66] Kosmrlj, A., E.L. Read, Y. Qi, T.M. Allen, M. Altfeld, S.G. Deeks, F. Pereyra, M. Carrington, B.D. Walker, and A.K. Chakraborty. 2010. Effects of thymic selection of the T-cell repertoire on HLA class I-associated control of HIV infection. Nature. 465:350–354. 10.1038/nature08997 20445539 PMC3098720

[bib67] Kurebayashi, S., E. Ueda, M. Sakaue, D.D. Patel, A. Medvedev, F. Zhang, and A.M. Jetten. 2000. Retinoid-related orphan receptor gamma (RORgamma) is essential for lymphoid organogenesis and controls apoptosis during thymopoiesis. Proc. Natl. Acad. Sci. USA. 97:10132–10137. 10.1073/pnas.97.18.10132 10963675 PMC27750

[bib68] Lancaster, J.N., Y. Li, and L.I.R. Ehrlich. 2018. Chemokine-mediated choreography of thymocyte development and selection. Trends Immunol. 39:86–98. 10.1016/j.it.2017.10.007 29162323 PMC5800975

[bib69] Lederberg, J. 1959. Genes and antibodies. Science. 129:1649–1653. 10.1126/science.129.3364.1649 13668512

[bib70] Lefranc, M.P. 2011. From IMGT-ONTOLOGY IDENTIFICATION axiom to IMGT standardized keywords: For immunoglobulins (IG), T cell receptors (TR), and conventional genes. Cold Spring Harb. Protoc. 2011:604–613. 10.1101/pdb.ip82 21632792

[bib71] Legoux, F.P., J.B. Lim, A.W. Cauley, S. Dikiy, J. Ertelt, T.J. Mariani, T. Sparwasser, S.S. Way, and J.J. Moon. 2015. CD4+ T cell tolerance to tissue-restricted self antigens is mediated by antigen-specific regulatory T cells rather than deletion. Immunity. 43:896–908. 10.1016/j.immuni.2015.10.011 26572061 PMC4654997

[bib72] Leonard, J.D., D.C. Gilmore, T. Dileepan, W.I. Nawrocka, J.L. Chao, M.H. Schoenbach, M.K. Jenkins, E.J. Adams, and P.A. Savage. 2017. Identification of natural regulatory T cell epitopes reveals convergence on a dominant autoantigen. Immunity. 47:107–117.e8. 10.1016/j.immuni.2017.06.015 28709804 PMC5562039

[bib73] Li, M.O., and A.Y. Rudensky. 2016. T cell receptor signalling in the control of regulatory T cell differentiation and function. Nat. Rev. Immunol. 16:220–233. 10.1038/nri.2016.26 27026074 PMC4968889

[bib74] Li, Q.J., J. Chau, P.J. Ebert, G. Sylvester, H. Min, G. Liu, R. Braich, M. Manoharan, J. Soutschek, P. Skare, . 2007. miR-181a is an intrinsic modulator of T cell sensitivity and selection. Cell. 129:147–161. 10.1016/j.cell.2007.03.008 17382377

[bib75] Liang, J., J. Lyu, M. Zhao, D. Li, M. Zheng, Y. Fang, F. Zhao, J. Lou, C. Guo, L. Wang, . 2017. Tespa1 regulates T cell receptor-induced calcium signals by recruiting inositol 1,4,5-trisphosphate receptors. Nat. Commun. 8:15732. 10.1038/ncomms15732 28598420 PMC5472764

[bib76] Liu, X., A. Adams, K.F. Wildt, B. Aronow, L. Feigenbaum, and R. Bosselut. 2003. Restricting Zap70 expression to CD4+CD8+ thymocytes reveals a T cell receptor-dependent proofreading mechanism controlling the completion of positive selection. J. Exp. Med. 197:363–373. 10.1084/jem.20021698 12566420 PMC2193832

[bib77] Lo, W.L., D.L. Donermeyer, and P.M. Allen. 2012. A voltage-gated sodium channel is essential for the positive selection of CD4(+) T cells. Nat. Immunol. 13:880–887. 10.1038/ni.2379 22842345 PMC3426661

[bib78] Lutes, L.K., Z. Steier, L.L. McIntyre, S. Pandey, J. Kaminski, A.R. Hoover, S. Ariotti, A. Streets, N. Yosef, and E.A. Robey. 2021. T cell self-reactivity during thymic development dictates the timing of positive selection. Elife. 10:e65435. 10.7554/eLife.65435 33884954 PMC8116051

[bib79] Ma, A., J.C. Pena, B. Chang, E. Margosian, L. Davidson, F.W. Alt, and C.B. Thompson. 1995. Bclx regulates the survival of double-positive thymocytes. Proc. Natl. Acad. Sci. USA. 92:4763–4767. 10.1073/pnas.92.11.4763 7761398 PMC41787

[bib80] Marrack, P., J.P. Scott-Browne, S. Dai, L. Gapin, and J.W. Kappler. 2008. Evolutionarily conserved amino acids that control TCR-MHC interaction. Annu. Rev. Immunol. 26:171–203. 10.1146/annurev.immunol.26.021607.090421 18304006 PMC3164820

[bib81] Mathis, D., and C. Benoist. 2009. Aire. Annu. Rev. Immunol. 27:287–312. 10.1146/annurev.immunol.25.022106.141532 19302042

[bib82] McCarron, M.J., M. Irla, A. Sergé, S.M. Soudja, and J.C. Marie. 2019. Transforming Growth Factor-beta signaling in αβ thymocytes promotes negative selection. Nat. Commun. 10:5690. 10.1038/s41467-019-13456-z 31857584 PMC6923358

[bib83] McDonald, B.D., J.J. Bunker, S.A. Erickson, M. Oh-Hora, and A. Bendelac. 2015. Crossreactive αβ T cell receptors are the predominant targets of thymocyte negative selection. Immunity. 43:859–869. 10.1016/j.immuni.2015.09.009 26522985 PMC4654978

[bib84] McGargill, M.A., and K.A. Hogquist. 1999. Antigen-induced coreceptor down-regulation on thymocytes is not a result of apoptosis. J. Immunol. 162:1237–1245. 10.4049/jimmunol.162.3.1237 9973375

[bib85] McNeill, L., R.J. Salmond, J.C. Cooper, C.K. Carret, R.L. Cassady-Cain, M. Roche-Molina, P. Tandon, N. Holmes, and D.R. Alexander. 2007. The differential regulation of Lck kinase phosphorylation sites by CD45 is critical for T cell receptor signaling responses. Immunity. 27:425–437. 10.1016/j.immuni.2007.07.015 17719247

[bib86] Messaoudi, I., J.A. Guevara Patiño, R. Dyall, J. LeMaoult, and J. Nikolich-Zugich. 2002. Direct link between mhc polymorphism, T cell avidity, and diversity in immune defense. Science. 298:1797–1800. 10.1126/science.1076064 12459592

[bib87] Michelson, D.A., K. Hase, T. Kaisho, C. Benoist, and D. Mathis. 2022. Thymic epithelial cells co-opt lineage-defining transcription factors to eliminate autoreactive T cells. Cell. 185:2542–2558.e18. 10.1016/j.cell.2022.05.018 35714609 PMC9469465

[bib88] Michelson, D.A., C. Zuo, M. Verzi, C. Benoist, and D. Mathis. 2023. Hnf4 activates mimetic-cell enhancers to recapitulate gut and liver development within the thymus. J Exp Med. 220:e20230461. 10.1084/jem.20230461 37399024 PMC10318407

[bib89] Moody, A.M., D. Chui, P.A. Reche, J.J. Priatel, J.D. Marth, and E.L. Reinherz. 2001. Developmentally regulated glycosylation of the CD8alphabeta coreceptor stalk modulates ligand binding. Cell. 107:501–512. 10.1016/s0092-8674(01)00577-3 11719190

[bib90] Morris, G.P., and P.M. Allen. 2012. How the TCR balances sensitivity and specificity for the recognition of self and pathogens. Nat. Immunol. 13:121–128. 10.1038/ni.2190 22261968 PMC13052442

[bib91] Muñoz-Rojas, A.R., and D. Mathis. 2021. Tissue regulatory T cells: Regulatory chameleons. Nat. Rev. Immunol. 21:597–611. 10.1038/s41577-021-00519-w 33772242 PMC8403160

[bib92] Murata, S., K. Sasaki, T. Kishimoto, S. Niwa, H. Hayashi, Y. Takahama, and K. Tanaka. 2007. Regulation of CD8+ T cell development by thymus-specific proteasomes. Science. 316:1349–1353. 10.1126/science.1141915 17540904

[bib93] Naramura, M., H.K. Kole, R.J. Hu, and H. Gu. 1998. Altered thymic positive selection and intracellular signals in Cbl-deficient mice. Proc. Natl. Acad. Sci. USA. 95:15547–15552. 10.1073/pnas.95.26.15547 9861006 PMC28080

[bib94] Oettinger, M.A., D.G. Schatz, C. Gorka, and D. Baltimore. 1990. RAG-1 and RAG-2, adjacent genes that synergistically activate V(D)J recombination. Science. 248:1517–1523. 10.1126/science.2360047 2360047

[bib95] Owen, R.D. 1945. Immunogenetic consequences of vascular anastomoses between bovine twins. Science. 102:400–401. 10.1126/science.102.2651.400 17755278

[bib96] Rajpal, A., Y.A. Cho, B. Yelent, P.H. Koza-Taylor, D. Li, E. Chen, M. Whang, C. Kang, T.G. Turi, and A. Winoto. 2003. Transcriptional activation of known and novel apoptotic pathways by Nur77 orphan steroid receptor. EMBO J. 22:6526–6536. 10.1093/emboj/cdg620 14657025 PMC291815

[bib97] Robins, H.S., S.K. Srivastava, P.V. Campregher, C.J. Turtle, J. Andriesen, S.R. Riddell, C.S. Carlson, and E.H. Warren. 2010. Overlap and effective size of the human CD8+ T cell receptor repertoire. Sci. Transl. Med. 2:47ra64. 10.1126/scitranslmed.3001442 PMC3212437 20811043

[bib98] Rocha, B., P. Vassalli, and D. Guy-Grand. 1991. The V beta repertoire of mouse gut homodimeric alpha CD8+ intraepithelial T cell receptor alpha/beta + lymphocytes reveals a major extrathymic pathway of T cell differentiation. J. Exp. Med. 173:483–486. 10.1084/jem.173.2.483 1824858 PMC2118783

[bib99] Rossjohn, J., S. Gras, J.J. Miles, S.J. Turner, D.I. Godfrey, and J. McCluskey. 2015. T cell antigen receptor recognition of antigen-presenting molecules. Annu. Rev. Immunol. 33:169–200. 10.1146/annurev-immunol-032414-112334 25493333

[bib100] Ruscher, R., R.L. Kummer, Y.J. Lee, S.C. Jameson, and K.A. Hogquist. 2017. CD8αα intraepithelial lymphocytes arise from two main thymic precursors. Nat. Immunol. 18:771–779. 10.1038/ni.3751 28530714 PMC5505317

[bib101] Saini, M., C. Sinclair, D. Marshall, M. Tolaini, S. Sakaguchi, and B. Seddon. 2010. Regulation of Zap70 expression during thymocyte development enables temporal separation of CD4 and CD8 repertoire selection at different signaling thresholds. Sci. Signal. 3:ra23. 10.1126/scisignal.2000702 20332428

[bib102] Sakaguchi, S., N. Mikami, J.B. Wing, A. Tanaka, K. Ichiyama, and N. Ohkura. 2020. Regulatory T cells and human disease. Annu. Rev. Immunol. 38:541–566. 10.1146/annurev-immunol-042718-041717 32017635

[bib103] Sant’Angelo, D.B., and C.A. Janeway Jr. 2002. Negative selection of thymocytes expressing the D10 TCR. Proc. Natl. Acad. Sci. USA. 99:6931–6936. 10.1073/pnas.102182499 12011450 PMC124506

[bib104] Savage, P.A., D.E.J. Klawon, and C.H. Miller. 2020. Regulatory T cell development. Annu. Rev. Immunol. 38:421–453. 10.1146/annurev-immunol-100219-020937 31990619

[bib105] Schatz, D.G., M.A. Oettinger, and D. Baltimore. 1989. The V(D)J recombination activating gene, RAG-1. Cell. 59:1035–1048. 10.1016/0092-8674(89)90760-5 2598259

[bib106] Sell, S., and P.G. Gell. 1965. Studies on rabbit lymphocytes in vitro. I. Stimulation of blast transformation with an antiallotype serum. J. Exp. Med. 122:423–440. 10.1084/jem.122.2.423 14316952 PMC2138061

[bib107] Sha, W.C., C.A. Nelson, R.D. Newberry, D.M. Kranz, J.H. Russell, and D.Y. Loh. 1988. Positive and negative selection of an antigen receptor on T cells in transgenic mice. Nature. 336:73–76. 10.1038/336073a0 3263574

[bib141] Shin, B., S.J. Chang, B.W. MacNabb, and E.V. Rothenberg. 2024. Transcriptional network dynamics in early T cell development. J. Exp. Med. 221. 10.1084/jem.2023089339167073

[bib108] Shinzawa, M., E.A. Moseman, S. Gossa, Y. Mano, A. Bhattacharya, T. Guinter, A. Alag, X. Chen, M. Cam, D.B. McGavern, . 2022. Reversal of the T cell immune system reveals the molecular basis for T cell lineage fate determination in the thymus. Nat. Immunol. 23:731–742. 10.1038/s41590-022-01187-1 35523960 PMC9098387

[bib109] Sim, B.C., L. Zerva, M.I. Greene, and N.R. Gascoigne. 1996. Control of MHC restriction by TCR valpha CDR1 and CDR2. Science. 273:963–966. 10.1126/science.273.5277.963 8688082

[bib110] Sin, J.H., J. Sucharov, S. Kashyap, Y. Wang, I. Proekt, X. Liu, A.V. Parent, A. Gupta, P. Kastner, S. Chan, . 2023. Ikaros is a principal regulator of Aire^+^ mTEC homeostasis, thymic mimetic cell diversity, and central tolerance. Sci. Immunol. 8:eabq3109. 10.1126/sciimmunol.abq3109 37889983 PMC11433069

[bib111] Singer, A., S. Adoro, and J.H. Park. 2008. Lineage fate and intense debate: Myths, models and mechanisms of CD4- versus CD8-lineage choice. Nat. Rev. Immunol. 8:788–801. 10.1038/nri2416 18802443 PMC2760737

[bib112] Sprent, J., and H. Kishimoto. 2002. The thymus and negative selection. Immunol. Rev. 185:126–135. 10.1034/j.1600-065X.2002.18512.x 12190927

[bib113] Stadinski, B.D., S.J. Blevins, N.A. Spidale, B.R. Duke, P.G. Huseby, L.J. Stern, and E.S. Huseby. 2019. A temporal thymic selection switch and ligand binding kinetics constrain neonatal Foxp3^+^ T_reg_ cell development. Nat. Immunol. 20:1046–1058. 10.1038/s41590-019-0414-1 31209405 PMC7039339

[bib114] Stadinski, B.D., S.B. Cleveland, M.A. Brehm, D.L. Greiner, P.G. Huseby, and E.S. Huseby. 2023. I-A^g7^ β56/57 polymorphisms regulate non-cognate negative selection to CD4^+^ T cell orchestrators of type 1 diabetes. Nat. Immunol. 24:652–663. 10.1038/s41590-023-01441-0 36807641 PMC10623581

[bib115] Stadinski, B.D., K. Shekhar, I. Gómez-Touriño, J. Jung, K. Sasaki, A.K. Sewell, M. Peakman, A.K. Chakraborty, and E.S. Huseby. 2016. Hydrophobic CDR3 residues promote the development of self-reactive T cells. Nat. Immunol. 17:946–955. 10.1038/ni.3491 27348411 PMC4955740

[bib116] Stadinski, B.D., P. Trenh, R.L. Smith, B. Bautista, P.G. Huseby, G. Li, L.J. Stern, and E.S. Huseby. 2011. A role for differential variable gene pairing in creating T cell receptors specific for unique major histocompatibility ligands. Immunity. 35:694–704. 10.1016/j.immuni.2011.10.012 22101158 PMC3253227

[bib117] Steier, Z., D.A. Aylard, L.L. McIntyre, I. Baldwin, E.J.Y. Kim, L.K. Lutes, C. Ergen, T.S. Huang, E.A. Robey, N. Yosef, and A. Streets. 2023. Single-cell multiomic analysis of thymocyte development reveals drivers of CD4^+^ T cell and CD8^+^ T cell lineage commitment. Nat. Immunol. 24:1579–1590. 10.1038/s41590-023-01584-0 37580604 PMC10457207

[bib118] Steier, Z., E.J.Y. Kim, D.A. Aylard, and E.A. Robey. 2024. The CD4 versus CD8 T cell fate decision: A multiomics-informed perspective. Annu Rev Immunol. 42:235–258. 10.1146/annurev-immunol-083122-040929 38271641

[bib119] Stepanek, O., A.S. Prabhakar, C. Osswald, C.G. King, A. Bulek, D. Naeher, M. Beaufils-Hugot, M.L. Abanto, V. Galati, B. Hausmann, . 2014. Coreceptor scanning by the T cell receptor provides a mechanism for T cell tolerance. Cell. 159:333–345. 10.1016/j.cell.2014.08.042 25284152 PMC4304671

[bib120] Stritesky, G.L., Y. Xing, J.R. Erickson, L.A. Kalekar, X. Wang, D.L. Mueller, S.C. Jameson, and K.A. Hogquist. 2013. Murine thymic selection quantified using a unique method to capture deleted T cells. Proc. Natl. Acad. Sci. USA. 110:4679–4684. 10.1073/pnas.1217532110 23487759 PMC3606987

[bib121] Sun, Z., D. Unutmaz, Y.R. Zou, M.J. Sunshine, A. Pierani, S. Brenner-Morton, R.E. Mebius, and D.R. Littman. 2000. Requirement for RORgamma in thymocyte survival and lymphoid organ development. Science. 288:2369–2373. 10.1126/science.288.5475.2369 10875923

[bib122] Surh, C.D., and J. Sprent. 1994. T-cell apoptosis detected in situ during positive and negative selection in the thymus. Nature. 372:100–103. 10.1038/372100a0 7969401

[bib123] Surh, C.D., and J. Sprent. 2008. Homeostasis of naive and memory T cells. Immunity. 29:848–862. 10.1016/j.immuni.2008.11.002 19100699

[bib124] Takahama, Y. 2006. Journey through the thymus: Stromal guides for T-cell development and selection. Nat. Rev. Immunol. 6:127–135. 10.1038/nri1781 16491137

[bib125] Talmage, D.W. 1957. Allergy and immunology. Annu. Rev. Med. 8:239–256. 10.1146/annurev.me.08.020157.001323 13425332

[bib126] Tarakhovsky, A., S.B. Kanner, J. Hombach, J.A. Ledbetter, W. Müller, N. Killeen, and K. Rajewsky. 1995. A role for CD5 in TCR-mediated signal transduction and thymocyte selection. Science. 269:535–537. 10.1126/science.7542801 7542801

[bib127] Taves, M.D., and J.D. Ashwell. 2021. Glucocorticoids in T cell development, differentiation and function. Nat. Rev. Immunol. 21:233–243. 10.1038/s41577-020-00464-0 33149283

[bib128] Thompson, J., and A. Winoto. 2008. During negative selection, Nur77 family proteins translocate to mitochondria where they associate with Bcl-2 and expose its proapoptotic BH3 domain. J. Exp. Med. 205:1029–1036. 10.1084/jem.20080101 18443228 PMC2373836

[bib129] Trowsdale, J., and J.C. Knight. 2013. Major histocompatibility complex genomics and human disease. Annu. Rev. Genomics Hum. Genet. 14:301–323. 10.1146/annurev-genom-091212-153455 23875801 PMC4426292

[bib130] Van Laethem, F., A.N. Tikhonova, L.A. Pobezinsky, X. Tai, M.Y. Kimura, C. Le Saout, T.I. Guinter, A. Adams, S.O. Sharrow, G. Bernhardt, . 2013. Lck availability during thymic selection determines the recognition specificity of the T cell repertoire. Cell. 154:1326–1341. 10.1016/j.cell.2013.08.009 24034254 PMC3792650

[bib131] von Boehmer, H., and H.J. Fehling. 1997. Structure and function of the pre-T cell receptor. Annu. Rev. Immunol. 15:433–452. 10.1146/annurev.immunol.15.1.433 9143695

[bib132] von Boehmer, H., and P. Kisielow. 2006. Negative selection of the T-cell repertoire: Where and when does it occur? Immunol. Rev. 209:284–289. 10.1111/j.0105-2896.2006.00346.x 16448549

[bib133] Vrisekoop, N., J.P. Monteiro, J.N. Mandl, and R.N. Germain. 2014. Revisiting thymic positive selection and the mature T cell repertoire for antigen. Immunity. 41:181–190. 10.1016/j.immuni.2014.07.007 25148022 PMC4152861

[bib134] Wang, A., J. Rud, C.M. Olson Jr., J. Anguita, and B.A. Osborne. 2009. Phosphorylation of Nur77 by the MEK-ERK-RSK cascade induces mitochondrial translocation and apoptosis in T cells. J. Immunol. 183:3268–3277. 10.4049/jimmunol.0900894 19675165

[bib135] Wirasinha, R.C., M. Singh, S.K. Archer, A. Chan, P.F. Harrison, C.C. Goodnow, and S.R. Daley. 2018. αβ T-cell receptors with a central CDR3 cysteine are enriched in CD8αα intraepithelial lymphocytes and their thymic precursors. Immunol. Cell Biol. 96:553–561. 10.1111/imcb.12047 29726044

[bib136] Xing, Q., D. Chang, S. Xie, X. Zhao, H. Zhang, X. Wang, X. Bai, and C. Dong. 2024. BCL6 is required for the thymic development of TCRαβ^+^CD8αα^+^ intraepithelial lymphocyte lineage. Sci. Immunol. 9:eadk4348. 10.1126/sciimmunol.adk4348 38335269

[bib137] Yagi, J., and C.A. Janeway Jr. 1990. Ligand thresholds at different stages of T cell development. Int. Immunol. 2:83–89. 10.1093/intimm/2.1.83 2150922

[bib138] Yamagata, T., D. Mathis, and C. Benoist. 2004. Self-reactivity in thymic double-positive cells commits cells to a CD8 alpha alpha lineage with characteristics of innate immune cells. Nat. Immunol. 5:597–605. 10.1038/ni1070 15133507

[bib139] Yang, S., N. Fujikado, D. Kolodin, C. Benoist, and D. Mathis. 2015. Immune tolerance. Regulatory T cells generated early in life play a distinct role in maintaining self-tolerance. Science. 348:589–594. 10.1126/science.aaa7017 25791085 PMC4710357

[bib140] Zinkernagel, R.M., and P.C. Doherty. 1974. Restriction of in vitro T cell-mediated cytotoxicity in lymphocytic choriomeningitis within a syngeneic or semiallogeneic system. Nature. 248:701–702. 10.1038/248701a0 4133807

